# Supplementary material for: Factors that influence the administration of tranexamic acid (TXA) to trauma patients in prehospital settings: a systematic review
Source: BMJ Open. 2023 May 31;13(5):e073075. doi: 10.1136/bmjopen-2023-073075 (PMC10255319; doi:10.1136/bmjopen-2023-073075)
Supplement: Supplementary data [file bmjopen-2023-073075supp001.pdf]

APPENDIX 1- Literature search report

Bibliographic database searches

Database: AMED

Host: EBSCO

Data Parameters: n/a

Date Searched: 10/1/2020

Searcher: SB

Hits: 2

Strategy:

1. TI "tranexamic acid" OR AB "tranexamic acid"
2. TI TXA OR AB TXA
3. TI cyklokapron OR AB cyklokapron
4. 1 or 2 or 3

Notes: Date limited 2010 – to date

Database: CENTRAL

Host: Cochrane Library

Data Parameters: Issue 1 of 12, January 2020

Date Searched: 10/10/2020

Searcher: SB

Hits: 196

Strategy:

#1 ("tranexamic acid"):ti OR ("tranexamic acid"):ab

#2 (TXA):ti OR (TXA):ab

#3 (cyklokapron):ti OR (cyklokapron):ab

#4 MeSH descriptor: [Tranexamic Acid] this term only

#5 #1 or #2 or #3 or #4

#6 (trauma*):ti OR (trauma*):ab

#7 (paramedic* or ems or emt or prehospital or "pre hospital" or "first responder*" or "emergency medical technician*" or "emergency services" or Ambulance* or HEMS or "field triage" or "out-of-hospital"):ti OR (paramedic* or ems or emt or prehospital or "pre hospital" or "first

responder*" or "emergency medical technician*" or "emergency services" or Ambulance* or HEMS or "field triage" or "out-of-hospital"):ab

#8 MeSH descriptor: [Ambulances] this term only

#9 MeSH descriptor: [Emergency Medical Technicians] this term only

#10 MeSH descriptor: [Air Ambulances] this term only

#11 MeSH descriptor: [Emergency Medical Services] this term only

#12 #6 or #7 or #8 or #9 or #10 or #11

#13 #5 and #12

Notes: Date limited 2010 – 2020

Database: CINAHL
Host: EBSCO
Data Parameters: n/a
Date Searched: 13/1/2020
Searcher: SB
Hits: 313
Strategy:

1. TI "tranexamic acid" OR AB "tranexamic acid"
2. TI TXA OR AB TXA
3. TI cyklokapron OR AB cyklokapron
4. (MH "Tranexamic Acid")
5. TI trauma* OR AB trauma*
6. TI ((paramedic* or ems or emt or prehospital or "pre hospital" or "first responder*" or "emergency medical technician*" or "emergency services" or Ambulance* or HEMS or "field triage" or "out-of-hospital")) OR AB ((paramedic* or ems or emt or prehospital or "pre hospital" or "first responder*" or "emergency medical technician*" or "emergency services" or Ambulance* or HEMS or "field triage" or "out-of-hospital"))
7. (MH "Ambulances")
8. (MH "Aeromedical Transport")
9. (MH "Emergency Medical Services+")
10. S1 OR S2 OR S3 OR S4
11. S5 OR S6 OR S7 OR S8 OR S9
12. S10 AND S11

Notes: Date limited 2010 – to date; English language results only

Database: Cochrane Database of Systematic Reviews (CDSR)

Host: Cochrane Library

Data Parameters: Issue 1 of 12, January 2020

Date Searched: 10/10/2010

Searcher: SB

Hits: 6

Strategy: see CENTRAL search

Notes: Date limited 2010 – 2020

Database: Conference Proceedings Citation Index – Science (CPCI-S)

Host: Clarivate Analytics

Data Parameters: n/a

Date Searched: 10/1/2020

Searcher: SB

Hits: 86

Strategy:

1. TOPIC: ("tranexamic acid")
2. TOPIC: (TXA)
3. TOPIC: (cyklokapron)
4. #1 OR #2 OR #3
5. TOPIC: (trauma*)
6. TOPIC: (paramedic* or ems or emt or prehospital or "pre hospital" or "first responder*" or "emergency medical technician*" or "emergency services" or Ambulance* or HEMS or "field triage" or "out-of-hospital")
7. #5 OR #6
8. (#7 AND #4) AND LANGUAGE: (English) Indexes=CPCI-S Timespan=2010-2020

Database: Embase

Host: Ovid

Data Parameters: 1974 to 2020 January 09

Date Searched: 10/1/2020

Searcher: SB

Hits: 1250

Strategy:

1. "tranexamic acid".tw.
2. TXA.tw.
3. cyklokapron.tw.
4. tranexamic acid/
5. or/1-4
6. trauma*.tw.
7. (paramedic* or ems or emt or prehospital or "pre hospital" or "first responder*" or "emergency medical technician*" or "emergency services" or Ambulance* or HEMS or "field triage" or "out-of-hospital").tw.
8. exp ambulance/
9. rescue personnel/
10. air medical transport/
11. exp emergency health service/
12. or/6-11
13. 5 and 12
14. limit 13 to (english language and yr="2010 –Current")

Database: MEDLINE ALL
Host: Ovid
Data Parameters: 1946 to January 09, 2020
Date Searched: 10/1/2020
Searcher: SB
Hits: 513
Strategy:

1. "tranexamic acid".tw.
2. TXA.tw.
3. cyklokapron.tw.
4. Tranexamic Acid/
5. or/1-4
6. trauma*.tw.
7. (paramedic* or ems or emt or prehospital or "pre hospital" or "first responder*" or "emergency medical technician*" or "emergency services" or Ambulance* or HEMS or "field triage" or "out-of-hospital").tw.
8. Ambulances/
9. Emergency Medical Technicians/
10. Air Ambulances/

11. Emergency Medical Services/
12. or/6-11
13. 5 and 12
14. Limit 13 to (english language and yr="2010 –Current")

Table A1. Total number of hits per database and unique records

Database	Hits
AMED	2
CENTRAL	196
CINAHL	313
CDSR	6
CPCI-S	86
Embase	1250
MEDLINE ALL	513
**Total records**	**2366**
**Duplicate records**	**605**
**Unique records**	**1761**

OpenGrey search

URL: http://www.opengrey.eu/

Date Searched: 09/03/2021

Searcher: HN

Hits: 11

Strategy: "tranexamic acid" OR TXA

Clinical trials registry search

Registry: ClinicalTrials.gov

URL: https://clinicaltrials.gov/

Date Searched: 24/06/2020

Searcher: HN

Hits: 27

Strategy:

Intervention/treatment: "tranexamic acid" OR TXA OR cyklokapron

Title/Acronym: trauma OR prehospital OR "pre-hospital" OR emergency

Update searches

Bibliographic database searches

Database: AMED

Host: EBSCO

Data Parameters: n/a

Date Searched: 14/12/2020

Searcher: SB

Hits: 0

Strategy: see above (date limited 2020 to date of search)

Database: CENTRAL

Host: Cochrane Library

Data Parameters: Issue 12 of 12, December 2020

Date Searched: 14/12/2020

Searcher: SB

Hits: 65

Strategy: see above (date limited 2020 to date of search)

Database: CINAHL

Host: EBSCO

Data Parameters: n/a

Date Searched: 14/12/2020

Searcher: SB

Hits: 68

Strategy: see above (date limited 2020 to date of search)

Database: Cochrane Database of Systematic Reviews (CDSR)

Host: Cochrane Library

Data Parameters: Issue 12 of 12, December 2020

Date Searched: 14/12/2020

Searcher: SB

Hits: 0

Strategy: see above (date limited 2020 to date of search)

Database: Conference Proceedings Citation Index – Science (CPCI-S)

Host: Clarivate Analytics

Data Parameters: n/a

Date Searched: 14/12/2020

Searcher: SB

Hits: 7

Strategy: see above (date limited 2020 to date of search)

Database: Embase

Host: Ovid

Data Parameters: 1974 to 2020 December 11

Date Searched: 14/12/2020

Searcher: SB

Hits: 227

Strategy: see above (date limited 2020 to date of search)

Database: MEDLINE ALL

Host: Ovid

Data Parameters: 1946 to December 11, 2020

Date Searched: 14/12/2020

Searcher: SB

Hits: 132

Strategy: see above (date limited 2020 to date of search)

Table A2. Total number of hits per database and unique records

**Database**	**Hits**
AMED	0
CENTRAL	65
CINAHL	68
CDSR	0
CPCI-S	7
Embase	227
MEDLINE ALL	132
**Total records**	**499**
**Duplicate records**	**189**
**Unique records**	**310**

Clinical trials registry search

Registry: ClinicalTrials.gov

URL: https://clinicaltrials.gov/

Date Searched: 25/01/2021

Searcher: HN

Hits: 30

Strategy: see above

[Please note that the hits reported from each of the clinical trials registry search updates included duplicates from the previous searches. However, we also found that there were both new records as well as records from previous searches that were now missing or had since been removed from the registry. Therefore, the number reported in the PRISMA diagram (34) was the total number of unique records found over the three searches.]

Update searches 2

Bibliographic database searches

Database: AMED

Host: EBSCO

Data Parameters: n/a

Date Searched: 8/6/2022

Searcher: SB

Hits: 4

Strategy: see above (date limited 2020 to date of search)

Database: CENTRAL

Host: Cochrane Library

Data Parameters: Issue 5 of 12, May 2022

Date Searched: 8/6/2022

Searcher: SB

Hits: 132

Strategy: see above (date limited 2020 to date of search)

Database: CINAHL

Host: EBSCO

Data Parameters: n/a

Date Searched: 9/6/2022

Searcher: SB

Hits: 712

Strategy: see above (date limited 2020 to date of search)

Database: Cochrane Database of Systematic Reviews (CDSR)

Host: Cochrane Library

Data Parameters: Issue 6 of 12, June 2022

Date Searched: 8/6/2022

Searcher: SB

Hits: 0

Strategy: see above (date limited 2020 to date of search)

Database: Conference Proceedings Citation Index – Science (CPCI-S)

Host: Clarivate Analytics

Data Parameters: n/a

Date Searched: 8/6/2022

Searcher: SB

Hits: 15

Strategy: see above (date limited 2020 to date of search)

Database: Embase

Host: Ovid

Data Parameters: 1974 to 2022 June 07

Date Searched: 8/6/2022

Searcher: SB

Hits: 571

Strategy: see above (date limited 2020 to date of search)

Database: MEDLINE ALL

Host: Ovid

Data Parameters: 1946 to June 07, 2022

Date Searched: 8/6/2022

Searcher: SB

Hits: 306

Strategy: see above (date limited 2020 to date of search)

Table A3. Total number of hits per database and unique records

Database	Hits
AMED	4
CENTRAL	132
CINAHL	712
CDSR	0
CPCI-S	15
Embase	571
MEDLINE ALL	306
**Total records**	**1740**
**Duplicate records**	**473**
**Duplicate record with previous search**	**222**
**Unique records**	**1045**

OpenGrey search

URL: http://www.opengrey.eu/

Date Searched: 25/01/2021

Searcher: HN

Hits: 18

Strategy: "tranexamic acid" OR TXA

Clinical trials registry search

Registry: ClinicalTrials.gov

URL: https://clinicaltrials.gov/

Date Searched: 25/01/2021

Searcher: HN

Hits: 33

Strategy: see above

[Please note that the hits reported from each of the clinical trials registry search updates included duplicates from the previous searches. However, we also found that there were both new records as well as records from previous searches that were now missing or had since been removed from the registry. Therefore, the number reported in the PRISMA diagram (34) was the total number of unique records found over the three searches.]

APPENDIX 2 - Inclusion and exclusion criteria

PEOS categories	Inclusion criteria	Exclusion criteria
**Population**	Any patients who have experienced traumatic injury	Patients receiving TXA who have not experienced traumatic injury or who have superficial minor wounds.<br><br>Patients experiencing obstetric, post-partum haemorrhage.
**Exposure**	Factors influencing the decision to administer TXA.	
**Outcome**	Papers detailing the characteristics of patients who did or did not receive TXA in the pre-hospital setting.<br><br>Papers detailing characteristics of pre-hospital clinicians who administered TXA.	TXA administration in hospital. Studies that solely report the clinical and/or cost effectiveness of TXA, dose or route of administration rather than the factors that influenced administration will be excluded.
**Setting**	Any pre-hospital setting where patients are treated by pre-hospital clinicians outside of a hospital or primary care environment.	Any hospital or primary care setting.
**Study design**	Any primary research.	Review articles, editorials and letters.

APPENDIX 3 – Variable sought during data extraction

List of variables for which data was sought during data extraction:

- Citation
- Country
- Study aims
- Patient and clinician population characteristics
- Primary outcome
- Inclusion and exclusion criteria
- Recruitment method
- Study Design
- Sample size and sampling method
- Data source
- Intervention and comparator group (if applicable)
- Date and duration of data collection
- Setting
- Analysis
- Factors influencing TXA administration

APPENDIX 4 – PRISMA diagram

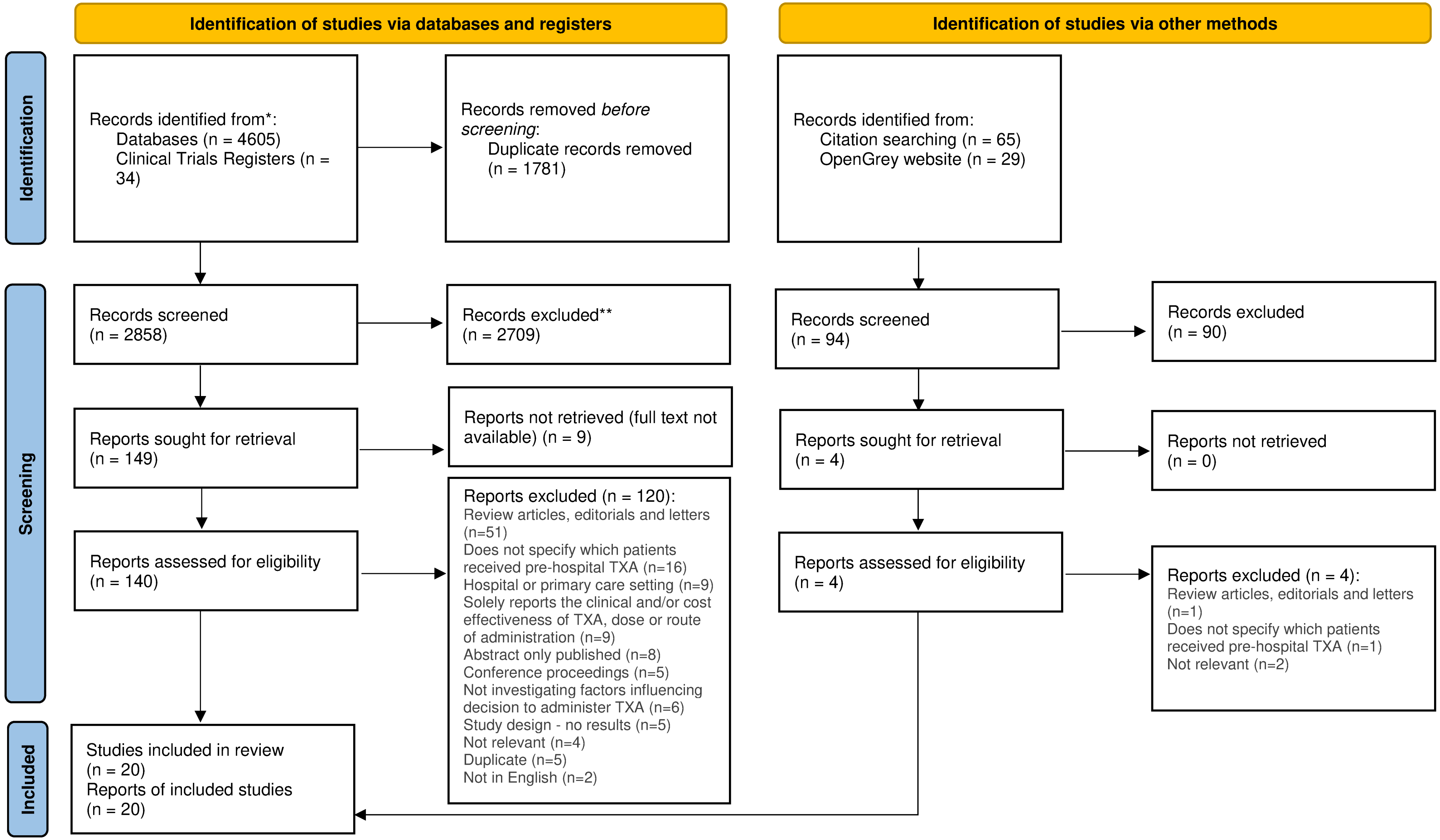

*Consider, if feasible to do so, reporting the number of records identified from each database or register searched (rather than the total number across all databases/registers).
**If automation tools were used, indicate how many records were excluded by a human and how many were excluded by automation tools.

From: Page MJ, McKenzie JE, Bossuyt PM, Boutron I, Hoffmann TC, Mulrow CD, et al. The PRISMA 2020 statement: an updated guideline for reporting systematic reviews. BMJ 2021;372:n71. doi: 10.1136/bmj.n71. For more information, visit: http://www.prisma-statement.org/

APPENDIX 5- Characteristics of included civilian studies (n=13).

First Author (date), country	Study design	Patient number	Data source	Dates and duration of data collection	Population	Factors influencing TXA administration
Bossers (2021), Netherlands	Observational mulitcentre cohort study	1827	Brain Injury: Prehospital Registry of Outcome, Treatments and Epidemiology of Cerebral Trauma (BRAINPROTECT) study database.	February 2012- December 2017, 5 years 10 months	Adult patients with severe TBI attended by HEMS	**Patient age** - patients receiving TXA older than those not receiving TXA (47 vs 45 years).<br><br>**Injury type, severity -** patients receiving TXA had higher Injury Severity Score (ISS) (27 vs 26), lower prehospital Glasgow Coma Score (GCS) (4 vs 5) and higher heart rate (98 vs 92).
El-Menyar (2020), Qatar	Retrospective observational, case control	204	Qatar National Trauma Registry	January 1 2017 to September 30 2018; 1 year 9 months	Adult trauma patients	**Resources** – patients did not receive TXA if critical care paramedics unavailable.
Goodwin (2021), UK	Qualitative interview study	18	UK paramedic interviews	2019	N/A	**Knowledge and skills** - inadequate training and a lack of knowledge of the effects of TXA or the evidence base behind its use and a lack of exposure to trauma patients were barriers to its administration.<br>**Resources -** a lack of time and staffing was a barrier to administration. Helicopter Emergency Medicine Services (HEMS) attendance was a barrier to some as they preferred to wait for a HEMS team member to administer it.<br>**Protocol -** guidelines felt restrictive or confusing. Disparity between paramedic, HEMS and doctor TXA protocols causes confusion. The drug preparation and administration route were seen as barriers to its use.<br>**Consequences and social influences-** the benefits of TXA were seen to outweigh the risks. TXA was seen to signal a major trauma patient. Fear of repercussion for administering TXA inappropriately. Opinion of colleagues seen to influence TXA administration.

						**Priorities** - TXA not seen as a priority, with administering fluids or distracting injuries often taking precedent. Three-hour window of administration may reduce the perceived urgency of administration. The stress associated with trauma jobs may lead to TXA being overlooked or deprioritised.<br>**Injury type, severity - r**isk of bleeding influenced administration including clinical observations and patient presentation. Participants found identifying patients at risk of bleeding difficult.<br>**Injury type, Mechanism of Injury (MOI) -** uncertainty over which injuries/conditions TXA is indicated for. MOI and type of injury were part of identify patients at risk of bleeding.<br>**Injury type, site** - less obvious bleeding including occult/internal haemorrhage was harder to identify.
Marsden (2019), UK	Retrospective service evaluation	661	TARN, local trauma registries and individual patient hospital records	January 1 2017 to December 31 2017; 1 year	Adult and paediatric trauma patients	**Injury type, MOI** – road traffic collisions more likely to receive TXA, falls less likely.<br>**Injury type, severity** – patients given TXA more likely to have heart rate and blood pressure suggestive of bleeding.<br>**Resources** – patients given TXA were more likely to be treated by a physician-led crew.
McQueen (2013), UK	Retrospective service evaluation	123	HEMS clinical database.	6 months - Dates not stated.	Major trauma patients attended by HEMS	**Protocol** - clinician judgement used to guide administration outside of the protocol.<br>**Injury type, MOI** – most TXA patients had multiple injuries.<br>**Resources** - time constraints on scene and the absence of a doctor as part of the HEMS crew meant TXA not given.
Neeki (2018), USA	Observational cohort study	724	Electronic medical record and trauma registry	March 2015 to July 2017; 2 years 4 months	Adult trauma patients	**Resources** - not all Emergency Medicine Service providers carrying TXA.
Ng (2018), Canada	Retrospective observational	117	British Columbia Trauma Registry and Royal Columbian Hospital records.	April 2012 to June 2015; 3 years 2 months.	Adult trauma patients	**Injury type, severity** – more patients receiving TXA had moderate (9-12) or severe (3-8) GCS scores than those not receiving TXA.
Nutbeam (2022), UK	Retrospective observational	216,364	Trauma and Audit Research	January 1 2017 to Dec		**Sex** - female patients less likely to receive TXA (OR 0.35, 95% CI, 0.33-0.36).

			Network (TARN) registry	31 2020; 4 years	Adult trauma patients	**Injury type, severity** - females less likely than males to receive TXA in all ISS categories.<br>**Injury type, MOI** - females less likely than males to receive TXA for all injury mechanisms except motor vehicle crashes.
van Wessem (2022), Netherlands	Prospective cohort study	234	Hospital data	November 2013; 7.5 years	Adult trauma patients with associated severe TBI	**Patient age** - patients receiving TXA younger than those not receiving TXA (42 vs 53 years).<br>**Injury type, severity -** patients needing pre-hospital intubation, urgent laparotomy or with more deranged physiology more likely to have received TXA.
van Wessem (2021), Netherlands	Population based Prospective cohort study	422	Hospital data	November 2013; 7 years	Adult trauma patients with associated severe TBI	**Patient age** - patients receiving TXA younger than those not receiving TXA (41 vs 51 years).<br>**Injury type, severity -** patients receiving TXA slightly more severely injured, had higher Abbreviated Injury Scale (AIS) Head scores and more often prehospitally intubated.
Vu (2013), USA	Case series	13	British Columbia Ambulance Service AirEvac and Critical Care Operation data system.	4 months - Dates unclear	Adult major trauma patients	**Priorities** - balancing critical interventions, resuscitation and short flight times meant some eligible patients did not receive TXA.
Wafaisade (2016), Germany	Retrospective observational	5765	ADAC Air Rescue Service prehospital database and German Trauma Society Trauma registry	January 1 2012 to December 31 2014; 3 years	Critically injured adult trauma patients	**Injury type, severity** - patients needing pre-hospital intubation or chest tube placement more likely to receive TXA.<br>**Injury type, site** - patients with AIS ≥3 for abdomen or extremities more likely to receive TXA. Patients with AIS ≥3 for head or thorax less likely to receive TXA.<br>**Age** - patients over 60 years old less likely to receive TXA.
Wong (2021), Canada	Retrospective observational	100	Hospital charts and British Columbia Trauma Registry	April 1 2016 to March 31 2017; 1 year	Adult trauma patients	**Age** - TXA group notably younger (38.2 years vs 49.1 years)<br>**Injury type, severity** - TXA group had higher mean ISS and more patients with hypotension.<br>**Resources** - more patients receiving TXA had a paramedic of higher certification level in attendance than those who did not receive TXA.

APPENDIX 6- Characteristics of included military setting studies (n=7).

First Author (date), country	Study design	Patient number	Data source	Dates and duration of data collection	Population	Factors influencing TXA administration
Benov (2019), Israel-Syrian Border	Retrospective observational	2339	Israel Defences Forces Trauma Registry	February 12 2013 to December 31 2017; 4 Years 10 months 19 days	Combat injuries, Syrian adult and paediatric refugees	**Protocol** – TXA for penetrating torso injury regardless of haemodynamic status. All patients receiving freeze-dried plasma with a known time of injury <3 hours received TXA.<br><br>**Injury type, MOI** – majority of TXA patients had penetrating injury.
Fisher (2019), Iraq and Afghanistan	Retrospective observational	28,222	The Department of Defence Trauma Registry.	January 2007 to August 2016; 9 years and 8 months.	Adult trauma patients	**Injury type, severity** – patients with higher ISS, tourniquet application or serious injuries to the thorax, abdomen, extremities and skin more likely to receive TXA.<br>**Injury type, MOI** - explosive injury and gunshot wounds were more likely to receive TXA. More likely to have explosive injuries and less likely gunshot wounds in TXA patients who had a tourniquet applied.<br>**Patient age** - amputation patients receiving TXA were younger than those not receiving TXA (22 vs 25 years).
Lipsky (2014), Israel	Retrospective observational	40	Israel Defences Forces Trauma Registry	December 2011 to February 2013; 1 year 2 months.	Adult trauma patients	**Protocol** – 30% of TXA administrations had no clear indication for TXA administration. Altered level of consciousness mistakenly categorised as a sign of haemodynamic instability.<br>**Priorities** – some non-administrations due to tactical limitations, resuscitation or to avoid delaying evacuation.
Mahalo (2021), Israel	Retrospective observational	1059	Israel Defences Force Trauma Registry and Israel National Trauma Registry	2006 to 2017; 10 years	Adult civilian and military trauma patients	**Injury type, severity** - TXA administration associated with analgesic treatment.
Nadler (2014), Israel	Retrospective observational	94	Israel Defences Forces Trauma Registry and hospital charts	December 2011 to August 2013; 1 year 8 months.	Adult civilian and military trauma patients	**Protocol** – more conservative protocol in the civilian service compared to the military service but higher proportion of patients given TXA outside of protocol (with clearance) by civilian service.

Nadler (2021), Israel	Retrospective observational	16529	Israel Defences Forces Trauma Registry and hospital charts	January 2006 to December 2018; 13 years	Adult civilian and military trauma patients	**Protocol -** New Clinical Practice Guidelines (indicating TXA at a heart rate of 130 instead of 110) introduced caused a significant decrease in the proportion of TXA administered. Only 22% of patients indicated for TXA received it.
Tsur (2020), Israel	Retrospective observational	3394	Israel Defences Forces Trauma Registry	January 1997 to December 2018; 11 years	Adult military trauma patients	**Injury type, site** – isolated neck injuries more likely to receive TXA than no-neck injuries.
