# Supplementary material for: Factors that influence the administration of tranexamic acid (TXA) to trauma patients in prehospital settings: a systematic review
Source: BMJ Open. 2023 May 31;13(5):e073075. doi: 10.1136/bmjopen-2023-073075 (PMC10255319; doi:10.1136/bmjopen-2023-073075)
Supplement: Author's manuscript [file bmjopen-2023-073075.draft_revisions.pdf]

BMJ Open

BMJ Open is committed to open peer review. As part of this commitment we make the peer review history of every article we publish publicly available.

When an article is published we post the peer reviewers' comments and the authors' responses online. We also post the versions of the paper that were used during peer review. These are the versions that the peer review comments apply to.

The versions of the paper that follow are the versions that were submitted during the peer review process. They are not the versions of record or the final published versions. They should not be cited or distributed as the published version of this manuscript.

BMJ Open is an open access journal and the full, final, typeset and author-corrected version of record of the manuscript is available on our site with no access controls, subscription charges or pay-per-view fees (http://bmjopen.bmj.com).

If you have any questions on BMJ Open's open peer review process please email info.bmjopen@bmj.com

BMJ Open

Factors that influence the administration of tranexamic acid (TXA) to trauma patients in pre-hospital settings: A systematic review.

Journal:	*BMJ Open*
Manuscript ID	bmjopen-2023-073075
Article Type:	Original research
Date Submitted by the Author:	23-Feb-2023
Complete List of Authors:	Nicholson, Helen; University of the West of England, College of Health, Science and Society<br>Scotney, Natalie; South Western Ambulance Service NHS Foundation Trust<br>Briscoe, Simon; University of Exeter, University of Exeter Medical School<br>Kirby, Kim; University of the West of England Department of Health and Social Sciences; South Western Ambulance Service NHS Foundation Trust, Research, Audit and Quality Improvement Department<br>Bedson, Adam; South Western Ambulance Service NHS Foundation Trust<br>Goodwin, Laura; University of the West of England, College of Health, Science and Society<br>Robinson , Maria; South Western Ambulance Service NHS Foundation Trust<br>Taylor, Hazel; University Hospitals Bristol NHS Foundation Trust, Research Design Service<br>Thompson Coon, Jo; University of Exeter, NIHR CLAHRC South West Peninsula<br>Voss, Sarah; University of the West of England, College of Health, Science and Society<br>Benger, Jonathan; University of the West of England, College of Health, Science and Society
Keywords:	ACCIDENT & EMERGENCY MEDICINE, TRAUMA MANAGEMENT, Systematic Review

SCHOLARONE™
Manuscripts

I, the Submitting Author has the right to grant and does grant on behalf of all authors of the Work (as defined in the below author licence), an exclusive licence and/or a non-exclusive licence for contributions from authors who are: i) UK Crown employees; ii) where BMJ has agreed a CC-BY licence shall apply, and/or iii) in accordance with the terms applicable for US Federal Government officers or employees acting as part of their official duties; on a worldwide, perpetual, irrevocable, royalty-free basis to BMJ Publishing Group Ltd ("BMJ") its licensees and where the relevant Journal is co-owned by BMJ to the co-owners of the Journal, to publish the Work in this journal and any other BMJ products and to exploit all rights, as set out in our licence.

The Submitting Author accepts and understands that any supply made under these terms is made by BMJ to the Submitting Author unless you are acting as an employee on behalf of your employer or a postgraduate student of an affiliated institution which is paying any applicable article publishing charge ("APC") for Open Access articles. Where the Submitting Author wishes to make the Work available on an Open Access basis (and intends to pay the relevant APC), the terms of reuse of such Open Access shall be governed by a Creative Commons licence – details of these licences and which Creative Commons licence will apply to this Work are set out in our licence referred to above.

Other than as permitted in any relevant BMJ Author's Self Archiving Policies, I confirm this Work has not been accepted for publication elsewhere, is not being considered for publication elsewhere and does not duplicate material already published. I confirm all authors consent to publication of this Work and authorise the granting of this licence.

For peer review only

Factors that influence the administration of tranexamic acid (TXA) to trauma patients in pre-hospital settings: A systematic review.

Helen Nicholson[1], Natalie Scotney[2], Simon Briscoe[3], Kim Kirby[1,2], Adam Bedson[2], Laura Goodwin[1], Maria Robinson[2], Hazel Taylor[4], Jo Thompson-Coon[5], Sarah Voss[1] & Jonathan Benger[1].

[1] College of Health, Science and Society, University of the West of England, Glenside Campus (1H14), Blackberry Hill, Bristol, BS16 1DD, England

[2] South Western Ambulance Service NHS Foundation Trust, Eagle Way, Exeter, EX2 7HY, England

[3] University of Exeter Medical School. University of Exeter, EX1 2LU

[4] Research Design Service – South West, University Hospitals Bristol NHS Foundation Trust, Upper Maudlin Street, Bristol, BS2 8AE, England

[5]NIHR Applied Research Collaboration South West Peninsula (PenARC), University of Exeter Medical School, University of Exeter, EX1 2LU

*corresponding author, email address: Helen5.Nicholson@uwe.ac.uk, Twitter: @EllisonHelen, ORCID ID: https://orcid.org/0000-0002-8964-0607

KEYWORDS: Tranexamic acid, emergency medical services, pre-hospital, hemorrhage, trauma management, systematic review.

ABSTRACT

Background: In the United Kingdom (UK) there are around 5,400 deaths annually from injury. Tranexamic acid (TXA) prevents bleeding and has been shown to reduce trauma mortality. TXA was introduced across UK Emergency Medical (ambulance) Services (EMS) during 2012, however only 5% of UK major trauma patients who are at risk of haemorrhage receive pre-hospital TXA. This systematic review aims to examine the evidence regarding factors influencing the administration of TXA to trauma patients.

Methods: We included all primary research reporting factors that influence TXA administration to patients with traumatic injury in the pre-hospital setting. We searched AMED, CENTRAL, CINAHL, Cochrane Database of Systematic Reviews, Conference Proceedings Citation Index – Science, Embase and MEDLINE from January 2010 to 2020; searches were updated in June 2022. We also searched clinicaltrials.gov and OpenGrey and performed forwards and backwards citation chasing. Two independent reviewers performed the selection process, quality assessment and data extraction. Data was tabulated, grouped by setting and influencing factor and synthesised narratively.

Results: Twenty papers were included in the final synthesis; 13 papers from civilian and 7 from military settings. Thirteen studies were rated as 'moderate' using the Effective Public Health Practice Project Quality Assessment Tool. Several common factors were identified: knowledge and skills; consequences and social influences; injury type (severity, injury site and mechanism); protocols; resources; priorities; patient age; patient sex.

Conclusions: This review highlights an absence of high-quality research. Preliminary evidence suggests a host of system and individual-level factors that may be important in determining whether TXA is administered to trauma patients in the pre-hospital setting.

For peer review only - http://bmjopen.bmj.com/site/about/guidelines.xhtml

Funding and registration: This review was supported by Research Capability Funding from the South Western Ambulance Service NHS Foundation Trust and the National Institute for Health Research Applied Research Collaboration South West Peninsula.
PROSPERO registration number CRD42020162943 (protocol available online).

STRENGTHS AND LIMITATIONS OF THIS STUDY

- Strengths of this review include the extensive searches, the use of best practice methods and tools, reporting according to PRISMA guidelines and the input of a multi-disciplinary study team.
- Generalisability was enhanced by the separation of civilian and military studies.
- The review was limited by the small number of relevant studies, which precluded a formal synthesis as well as an English language restriction (two studies excluded due to language).
- In a deviation from the protocol, we were unable to search the ICTRP Search Portal. However, we were able to search the ClinicalTrials.gov registry which did not identify any new studies for inclusion; we therefore believe the impact of this was minimal.

INTRODUCTION

At least 20,000 individuals are severely injured in England each year, resulting in approximately 5,400 deaths, of which 2,400 occur before hospital arrival[1]. The CRASH2 trial provided strong evidence of the effectiveness of tranexamic acid (TXA) in reducing mortality in bleeding trauma patients[2], with CRASH3 then extending its use to isolated head injuries[3]. When given within three hours of injury, TXA reduces death from bleeding in all types of trauma[4]. An additional exploratory analysis concluded that the intervention is more effective if administered as soon as possible, particularly within one hour[5]. In their meta-analysis of the CRASH2 and WOMAN trials, Gayet-Ageron et al. found that immediate treatment improved survival by more than 70%, and that the survival benefit decreased by 10% for every 15 minutes of delay[6].

In 2011 South Western Ambulance Service NHS Foundation Trust was the first Emergency Medical Services (EMS) Provider (known as ambulance services in England) to implement the recommendations of CRASH2[7]; TXA was subsequently introduced across all UK ambulance services. However, analysis of UK Trauma Audit and Research Network (TARN) data found that only 5% of UK major trauma patients who are at risk of haemorrhage receive pre-hospital TXA[8]. The percentage of patients given TXA within one hour varies, with a reported range of 30% to 59%[9,10].

The timing of TXA administration is the single factor that determines effectiveness,[4,5] and the key determinant of time to administration is the setting in which it is given[10]. TARN data show that when administered pre-hospital, median time to TXA administration is 49 minutes, compared to a median time of 111 minutes when given in hospital[10].

Determining the full extent of a patient's injuries, and the associated bleeding risk, in the early stages of pre-hospital care can be challenging. This systematic review aims to examine evidence regarding factors that influence the timely administration of TXA to pre-hospital trauma patients. Due to the paucity of literature on this subject from UK civilian pre-hospital practice this review included both international and military-based pre-hospital care in order to maximise the identification of potential influencing factors. This approach was intended to give the greatest possible insights into TXA administration.

METHODS

Searches

We searched: AMED (via EBSCO); CENTRAL (via the Cochrane Library); CINAHL (via EBSCO); Cochrane Database of Systematic Reviews (via the Cochrane Library); Conference Proceedings Citation Index – Science (via Web of Science); Embase (via Ovid); and MEDLINE (via Ovid). The search strategy was developed using MEDLINE by an information specialist (SB) in consultation with the team. It included a sensitivity maximising search filter for identifying studies on EMS staff (paramedics)[11] and the final version was tested using pre-identified studies to ensure it retrieved all known relevant research. Searches were conducted on 10/01/2020 with a date limit of 2010 (reflecting the publication date of CRASH-2 [2], and most recently updated on 08/06/2022. Search results were exported to Endnote X8 and de-duplicated. The search strategies for each bibliographic database are reported in Appendix 1.

We supplemented these searches using ClinicalTrials.gov (see Appendix 1 for search terms), checking reference lists, forward citation searching and OpenGrey (www.opengrey.eu). Forward citation searching used the Science Citation Index (via Web of Science) on 07/07/2020, 29/01/2021 and 13/12/2022. If an included study was not indexed in the Science Citation Index we used either Scopus or Google Scholar. Reference lists of included studies were checked manually.

Article selection

All primary pre-hospital research involving trauma patients and detailing any factors influencing TXA administration was included (see Appendix 2 for inclusion and exclusion criteria). Due to resource constraints the review was limited to English language publications. Study titles and abstracts were screened by two independent reviewers (HN and NK/KK). Disagreements were resolved through arbitration by a third reviewer (SV). The full text of all articles that appeared to meet eligibility criteria were retrieved. Full texts were assessed following the same process as the title and abstract screening. All eligible papers proceeded to data extraction.

Quality Appraisal

The Quality Assessment Tool for Quantitative Studies from the Effective Public Health Practice Project (EPHPP) was independently used by two reviewers (HN and NK/KK) to assess quantitative studies for risk of bias, study design, confounders and results[12,13]. Studies were graded 1-3 (strong, moderate or weak). Qualitative studies were assessed using the Critical Appraisal Skills Programme (CASP) Qualitative Research Checklist [14].

Studies were not excluded on the basis of poor quality, but methodological limitations and their effect on confidence in the findings of each paper were considered when drawing conclusions[15].

Data collection and synthesis

Data was extracted by two independent reviewers (HN and NK/KK) using a pre-determined data collection table (see Appendix 3 for data collection variables). Studies were tabulated and grouped by influencing factors and described with the aim of identifying patterns.

RESULTS

For peer review only

The PRISMA diagram is shown in Appendix 4. Twenty papers were included in the final synthesis. In order to aid interpretation, the papers were divided into civilian (13 papers) and military settings (7 papers) (Table 1 & Appendix 5 and Table 2 & Appendix 6 respectively). All but one of the papers were quantitative in design. Thirteen of the studies were retrospective observational or service evaluation studies, with four observational cohort studies [16–19], one case control study[20], one case series [21] and one qualitative interview study [22].

Thirteen studies were rated as 'moderate' using the EPHPP Quality Assessment Tool. One study was rated 'strong'[20] due to its case-control design. One abstract[23] was rated 'weak' due to a lack of published information and four other papers were rated 'weak' as a result of study design and data collection methods [17–19,24] . The qualitative study was rated as 'valuable' using the CASP Qualitative Research Checklist [14]. All but five of the studies were retrospective, and data collection was often incomplete. However, all of the studies explained patient dropouts or exclusions except the abstract[23].

Only two of the papers included paediatric patients in their studies[9,25] and three of the papers limited their studies to adult trauma patients with severe traumatic brain injury[17–19]. Only the Goodwin et al. paper had primary outcomes relating directly to factors influencing the pre-hospital administration of TXA[22]. This was a qualitative study interviewing 18 UK paramedics from one ambulance service about the barriers and facilitators of TXA administration. Using the factors reported from this paper and the reported outcomes from the other papers it was possible to identify several factors of interest: knowledge and skills; consequences and social influences; injury type (including severity, injury site and mechanism of injury); protocols; resources; priorities; patient age; patient sex.

For peer review only

Table 1. Summary table of characteristics of included civilian studies (n=13) (see Appendix 5 for full table).

First Author (date), country	Factors influencing TXA administration
Bossers (2021), Netherlands	**Patient age** - patients receiving TXA older than those not receiving TXA (47 vs 45 years).<br>**Injury type, severity -** patients receiving TXA had higher Injury Severity Score (ISS) (27 vs 26), lower prehospital Glasgow Coma Score (GCS) (4 vs 5) and higher heart rate (98 vs 92).
El-Menyar (2020), Qatar	**Resources** – patients did not receive TXA if critical care paramedics unavailable.
Goodwin (2021), UK	**Knowledge and skills** - inadequate training and a lack of knowledge of the effects of TXA or the evidence base behind its use and a lack of exposure to trauma patients were barriers to its administration.<br>**Resources -** a lack of time and staffing was a barrier to administration. Helicopter Emergency Medicine Services (HEMS) attendance was a barrier to some as they preferred to wait for a HEMS team member to administer it.<br>**Protocol -** guidelines felt restrictive or confusing. Disparity between paramedic, HEMS and doctor TXA protocols causes confusion. The drug preparation and administration route were seen as barriers to its use.<br>**Consequences and social influences-** the benefits of TXA were seen to outweigh the risks. TXA was seen to signal a major trauma patient. Fear of repercussion for administering TXA inappropriately. Opinion of colleagues seen to influence TXA administration.<br>**Priorities** - TXA not seen as a priority, with administering fluids or distracting injuries often taking precedent. Three-hour window of administration may reduce the perceived urgency of administration. The stress associated with trauma jobs may lead to TXA being overlooked or deprioritised.<br>**Injury type, severity - r**isk of bleeding influenced administration including clinical observations and patient presentation. Participants found identifying patients at risk of bleeding difficult.<br>**Injury type, Mechanism of Injury (MOI) -** uncertainty over which injuries/conditions TXA is indicated for. MOI and type of injury were part of identify patients at risk of bleeding.<br>**Injury type, site** - less obvious bleeding including occult/internal haemorrhage was harder to identify.
Marsden (2019), UK	**Injury type, MOI** – road traffic collisions more likely to receive TXA, falls less likely.<br>**Injury type, severity** – patients given TXA more likely to have heart rate and blood pressure suggestive of bleeding.<br>**Resources** – patients given TXA were more likely to be treated by a physician-led crew.
McQueen (2013), UK	**Protocol** - clinician judgement used to guide administration outside of the protocol.<br>**Injury type, MOI** – most TXA patients had multiple injuries.<br>**Resources** - time constraints on scene and the absence of a doctor as part of the HEMS crew meant TXA not given.
Neeki (2018), USA	**Resources** - not all Emergency Medicine Service providers carrying TXA.
Ng (2018), Canada	**Injury type, severity** – more patients receiving TXA had moderate (9-12) or severe (3-8) GCS scores than those not receiving TXA.

For peer review only

Nutbeam (2022), UK	**Sex** - female patients less likely to receive TXA (OR 0.35, 95% CI, 0.33-0.36).<br>**Injury type, severity** - females less likely than males to receive TXA in all ISS categories.<br>**Injury type, MOI** - females less likely than males to receive TXA for all injury mechanisms except motor vehicle crashes.
van Wessem (2022), Netherlands	**Patient age** - patients receiving TXA younger than those not receiving TXA (42 vs 53 years).<br>**Injury type, severity -** patients needing pre-hospital intubation, urgent laparotomy or with more deranged physiology more likely to have received TXA.
van Wessem (2021), Netherlands	**Patient age** - patients receiving TXA younger than those not receiving TXA (41 vs 51 years).<br>**Injury type, severity -** patients receiving TXA slightly more severely injured, had higher Abbreviated Injury Scale (AIS) Head scores and more often prehospitally intubated.
Vu (2013), USA	**Priorities** - balancing critical interventions, resuscitation and short flight times meant some eligible patients did not receive TXA.
Wafaisade (2016), Germany	**Injury type, severity** - patients needing pre-hospital intubation or chest tube placement more likely to receive TXA.<br>**Injury type, site** - patients with AIS ≥3 for abdomen or extremities more likely to receive TXA. Patients with AIS ≥3 for head or thorax less likely to receive TXA.<br>**Age** - patients over 60 years old less likely to receive TXA.
Wong (2021), Canada	**Age** - TXA group notably younger (38.2 years vs 49.1 years)<br>**Injury type, severity** - TXA group had higher mean ISS and more patients with hypotension.<br>**Resources** - more patients receiving TXA had a paramedic of higher certification level in attendance than those who did not receive TXA.

Table 2. Summary table of characteristics of included military setting studies (n=7). (see Appendix 6 for full table).

First Author (date), country	Factors influencing TXA administration
Benov (2019), Israel-Syrian Border	**Protocol** – TXA for penetrating torso injury regardless of haemodynamic status. All patients receiving freeze-dried plasma with a known time of injury <3 hours received TXA.<br>**Injury type, MOI** – majority of TXA patients had penetrating injury.
Fisher (2019), Iraq and Afghanistan	**Injury type, severity** – patients with higher ISS, tourniquet application or serious injuries to the thorax, abdomen, extremities and skin more likely to receive TXA.<br>**Injury type, MOI** - explosive injury and gunshot wounds were more likely to receive TXA. More likely to have explosive injuries and less likely gunshot wounds in TXA patients who had a tourniquet applied.<br>**Patient age** - amputation patients receiving TXA were younger than those not receiving TXA (22 vs 25 years).
Lipsky (2014), Israel	**Protocol** – 30% of TXA administrations had no clear indication for TXA administration. Altered level of consciousness mistakenly categorised as a sign of haemodynamic instability.<br>**Priorities** – some non-administrations due to tactical limitations, resuscitation or to avoid delaying evacuation.

For peer review only

Mahalo (2021), Israel	**Injury type, severity** - TXA administration associated with analgesic treatment.
Nadler (2014), Israel	**Protocol** – more conservative protocol in the civilian service compared to the military service but higher proportion of patients given TXA outside of protocol (with clearance) by civilian service.
Nadler (2021), Israel	**Protocol -** New Clinical Practice Guidelines (indicating TXA at a heart rate of 130 instead of 110) introduced caused a significant decrease in the proportion of TXA administered. Only 22% of patients indicated for TXA received it.
Tsur (2020), Israel	**Injury type, site** – isolated neck injuries more likely to receive TXA than no-neck injuries.

For peer review only

Injury type

This factor was the most prevalent (14/19 papers)[9,23,25–29] [17–19,22,24,30,31] and could be further divided into injury severity, injury site and mechanism of injury.

Injury severity: Patients with a higher Injury Severity Score (ISS) were more likely to receive TXA in both military and civilian settings[17,24,26]. This was also the case within various sub-cohorts of combat casualties, such as those who had a tourniquet applied or with gunshot wounds[26]. Civilian patients needing pre-hospital tracheal intubation [18,19] or chest tube placement were also more likely to receive TXA[29]. However, one paper found no difference in ISS between patients that received pre-hospital and in-hospital TXA[9]. Mahalo et al. found that TXA administration in combat casualties was associated with analgesic treatment[30].

Injury site: Injury location influenced TXA administration, with paramedics reporting that less obvious bleeding, such as internal haemorrhage, was harder to identify therefore making the administration of TXA less likely[22]. Combat casualties were more likely to receive TXA if they suffered an isolated neck injury (15%, compared to 4% without neck injuries: P=0.01)[28]. This may be due, in part, to the fact that the neck is mostly a non-compressible area, and therefore requires alternative haemorrhage control strategies. However, the number of isolated neck injury patients was small (41, compared to 3,185 without neck injuries over a 12-year period).

In one civilian study, when using the Abbreviated Injury Score (AIS) to assess trauma patients, a score of 3 or more for the head or thorax was associated with a significantly lower chance of receiving TXA, whereas for the abdomen or extremities it was associated with an increased chance[29]. However, van Wessem et al. found that patients receiving TXA had higher Abbreviated Injury Scale (AIS) Head scores[19].

Ng et al. found that more patients receiving TXA in a civilian setting had a moderately or severely reduced Glasgow Coma Scale (GCS) score compared to those not receiving TXA, although statistical significance was not reported[27]. This was supported by Bossers et al. who found that patients receiving TXA had a significantly lower pre-hospital GCS than those not receiving it[17].

Mechanism of injury: Patients with multiple injuries made up 72% of patients receiving TXA in one study[23]. Mechanism of injury influenced the likelihood of receiving pre-hospital TXA in some of the civilian studies. Marsden et al. compared those receiving pre-hospital TXA with those receiving it in hospital[9]. Mechanism of injury was a statistically significant factor, with pre-hospital clinicians more likely to recognise the need for TXA in road traffic collisions than in falls. Penetrating injury was a positive determinant of TXA administration even when protocols did not specifically prompt

this[9,20]. Penetrating trauma was also linked to TXA administration in combat casualties,[26,32] with patients sustaining explosive injury and gunshot wounds more likely to receive TXA [26]. Participants in the Goodwin et al. study confirmed that mechanism of injury played a part in identifying patients at risk of bleeding, however, they also reported uncertainty over which injuries or conditions TXA was indicated for [22].

Protocols

This factor encompassed both the contents of protocols (indications for use, route of administration) and the how they were used (compliance, confusion) and it was closely linked to injury type. Where described, the details of TXA protocols varied, but usually included exact indications (patient observation ranges and/or mechanism of injury)[18–21,24,25,32–34]. Recognised signs of clinical shock were most often the basis of these protocols[18–21,33]. In some, the presence of major trauma[21], the use of freeze-dried plasma[25], capillary refill time or altered level of consciousness were also included[33]. However, for the National Israeli Civilian Services only age (>18 years), expected evacuation times (>15 minutes) and the presence of a non-compressible haemorrhage were specified as indications[32]. The Tactical Combat Casualty Care guidelines used by the US military employ the more subjective indication of "anticipated to need significant blood transfusion"[26]. This was similar to the "clinical suspicion of major haemorrhage" or "clinical judgement" reported in the indications for TXA in some civilian studies[18,19,23].

This variation in protocol was also mentioned by participants in the Goodwin et al. study where they felt that the disparity between paramedic, helicopter emergency medical services (HEMS) and doctor TXA protocols caused confusion[22]. They reported that the guidelines for TXA use felt restrictive or confusing, and that the drug preparation and administration route were seen as barriers to its use.

The TXA protocol for the Israel Defence Force specifies use in patients receiving freeze-dried plasma as well as those with penetrating torso injury, regardless of haemodynamic status[25]. It is therefore unsurprising that 50.9% (n=113) who received TXA in the Benov et al. study presented with penetrating injury and tachycardia, and all patients known to be less than three hours from injury who received freeze-dried plasma also received TXA[25]. However, this study was limited by incomplete data collection and the assumption that severely injured patients would likely have died before reaching medical assistance.

Resources

Time, clinician and TXA availability appear to influence TXA administration[9,16,20,22,23]. In addition to twelve cases (48%) of TXA administrations being outside protocol, McQueen & Wyse noted a further thirteen (46%) indicated cases where TXA was not administered[23]. They identified time constraints on scene and the absence of a doctor as reasons for non-administration. El-Menyar et al. found that the time from EMS activation to hospital arrival was significantly longer in the TXA group (62 vs 74 minutes, p=0.03)[20]. This suggests that TXA administration is associated with increased pre-hospital time; either due to the requirements of drug administration, or additional procedures that patients who are given TXA require at scene.

The grade of clinician able to administer TXA varied. El-Menyar et al. reported that TXA could only be administered by critical care paramedics, and suggested that a lack of availability of these staff led to some eligible patients not receiving TXA[20]. Marsden et al. found that patients who were given TXA were more likely to be treated by a physician-led crew (60.5% vs 31.6%, p<0.001)[9] and Wong et al. found that patients receiving TXA were attended by a paramedic of higher certification level than

For peer review only

those who did not receive TXA[24]. However, some participants in the Goodwin et al. study reported that HEMS attendance was a barrier to TXA administration as they preferred to wait for a HEMS team member to administer it[22].

However, the most fundamental resource issue is the availability of the drug itself, and despite strong evidence of effectiveness some services did not carry TXA[16].

Priorities

The Goodwin et al. study found that TXA was not seen as a priority by the paramedics interviewed with fluid administration or the treatment of injuries often taking precedent[22]. Some studies also suggested that other considerations may take priority over TXA administration. For example, Vu et al. found several patients who met the criteria but did not receive TXA, although the exact number was not disclosed[21]. In these cases, clinicians reported other priorities that required their attention as they balanced critical interventions, resuscitation and short flight times[21] A similar situation was described by Lipsky et al. who found that some non-administrations may have been due to tactical limitations, resuscitation or to avoid delaying evacuation[33]. However, they also suggested that raising awareness amongst staff may prove beneficial.

The participants in the Goodwin et al. study also suggested that the 3-hour window may reduce the perceived urgency of TXA administration and that the stress associated with trauma patients may lead to TXA being overlooked or deprioritised[22].

Patient age

There was some evidence that a patient's age influenced administration, with patients over 60 years less likely to receive TXA in one study[29] and patients receiving TXA being notably younger (38.2 years vs 49.1 years) than those who did not in another study[24]. The van Wessem studies also found that trauma patients with severe traumatic brain injury receiving TXA were younger than those who did not (42 vs 53 years and 41 vs 51 years), although the seven years of data from the 2021 study were reused but extended by 6 months in the 2022 study meaning these should not be considered separate results[18,19]. However, Bossers et al. found that patients with severe traumatic brain injury receiving TXA were slightly older than those not receiving it (47 vs 45 years)[17].

In military settings Fisher et al. found that, of those with amputations, patients receiving TXA were younger than those not receiving TXA (22 vs 25 years, $p<0.001$)[26], though some of these differences are of questionable clinical significance.

Patient sex

Nutbeam et al. was the only paper to report a patient sex difference in TXA administration[31]. They found that female patients in all risk categories and age groups were less likely to receive TXA (OR 0.35, 95% CI, 0.33-0.36). This difference in receipt of TXA increased with increasing age and decreased as injury severity increased. Females were found to be less likely to receive TXA for all mechanisms of injury except motor vehicle crashes.

Knowledge and Skills

The participants in the Goodwin et al. study reported that inadequate training and a lack of knowledge of the effects of TXA or the evidence base behind its use were barriers to its administration[22]. A lack of exposure to trauma patients was also reported as a barrier to its administration.

Consequences and social influences

Paramedics interviewed in the Goodwin et al. study felt that the benefits of TXA outweigh the risks[22]. However, administering TXA was seen to signal a major trauma patient, leading to concerns about the acceptance of the patient at a major trauma centre or judgement from colleagues if it was given to less severely injured patients. The opinion of colleagues was seen as an influencing factor, with 7/18 paramedics reporting that they check with a senior colleague or hospital staff before administering TXA. Paramedics reported a fear of repercussion for administering TXA inappropriately. They also felt that a lack of visible effects on patients inhibited TXA use.

DISCUSSION

This review found a paucity of high-quality research addressing the factors that influence pre-hospital TXA administration. However, despite only one of the included papers having primary outcomes relating directly to factors influencing the pre-hospital administration of TXA, it was possible to identify several factors from the studies that suggested a host of system and individual-level factors that may be important in determining whether TXA is administered to trauma patients in the pre-hospital setting. Injury type and protocols were the most prominent factors.

Despite the fact that this systematic review is the most complete to date of factors influencing pre-hospital TXA administration, there are some limitations. Only two of the studies[9,25] included paediatric patients, and the number of children in each were low. Therefore, it is not possible to identify factors influencing the administration of TXA to children in this review. There was also variability between the protocols and resources available to pre-hospital clinicians as well as settings in the included studies.

Seven of the papers reported on patients from combat settings[25,26,28,30,32–34]. Although much has been learnt about trauma care from military medicine, these studies are less likely to be generalisable to civilian trauma, and included disproportionately high numbers of young men and penetrating trauma. Despite this, the influencing factors identified were in line with those found in the civilian studies. However, in the military studies protocol and injury type seemed to play a more prominent role. Generalisability of the review was aided by the separation of civilian and military studies. Of the seven combat-specific studies, six were based on the experiences of the same pre-hospital service and included considerable author, data and date range overlap. A similar issue was found for the two studies from the Netherlands [18,19]. This raises the issue of potential pseudo-replication within the review. However, strengths of this review include the extensive searches, the use of PRISMA methods and tools and the input of a multi-disciplinary study team.

Protocols played an understandably important role in TXA administration[25]. However, criteria for administration and protocol compliance varied. In general protocol compliance was poor, suggesting that alternative factors play a part in clinical decision-making. This variability in protocol compliance results in both under administration and inappropriate administration of TXA[9,24,27,34–36]. Goodwin et al. found that confusion and restrictions relating to guidelines for TXA administration were barriers to administration[22]. Lipsky et al. reported that 30% of TXA administrations were for patients with no clear indication[33]. They found that in some of these cases altered levels of consciousness from blunt head injury appeared to be mistaken for haemodynamic instability. McQueen & Wyse reported that 48% of TXA administrations did not fulfil the inclusion criteria in their protocol[23], which they attributed to the influence of clinician judgement. However, a more subjective guideline relying on a clinician's ability to identify patients likely to require significant

For peer review only - http://bmjopen.bmj.com/site/about/guidelines.xhtml

blood transfusion resulted in only 4.2% of those given TXA receiving ≥10 units of blood within the following 24 hours[26]. This suggests that clinical judgement may not be the most effective approach to guiding TXA administration, given that it can result in both low administration rates and high levels of inappropriate use.

It therefore seems preferable to focus on more objective protocols combined with improved compliance. Nadler et al. found that only 22% of patients indicated for TXA in a military setting received it[34]. In a civilian setting Wong et al. found that 35% of eligible patients had received pre-hospital TXA and reported that this was higher than other rates reported in Canada[24]. They suggested that factors such as poor intra-venous accessibility, time constraints or short transport times might limit willingness to administer TXA. Naddler et al. suggested that significant over and under treatment of TXA may indicate insufficient professionalism or insufficient knowledge of the practice guidelines[34]. Other papers suggested ways to improve compliance including raising awareness, improved training and the use of a decision-making tool to aid in identifying a patient's bleeding risk[22,33]. However, the inclusion of training on TXA administration is not always mandated, even within combat medical training, and compliance with training can be low[26].

The most frequent factors mentioned in protocols were signs of clinical shock, penetrating injury and administration of other treatments. It is not surprising that patients who receive pre-hospital TXA tend to have more overt haemodynamic instability[9]. This may be a product of pre-hospital protocols that tend to be weighted towards signs of clinical shock as a key criterion for TXA administration.

The lack of obvious benefits at the time of administration was suggested as a relevant factor in the study by Goodwin et al.[22]. Interestingly, Lipsky et al. included the fact that survival benefit is only seen after 24-48 hours in their protocol training materials[33]. They suggest informing clinicians that they should not expect improved field haemorrhage control following TXA. It is possible that making this clear in training materials or protocols may help to address one potential barrier to TXA administration. Another factor that may contribute to low administration rates is the need for slow intravenous infusion of TXA. Clinicians in the Fisher et al. study were required to administer TXA using a 10-minute infusion[26]. The authors suggest this may not be compatible with a tactical situation and propose that oral or intramuscular TXA may help to increase administration rates. The requirement for slow intravenous administration was also found to be a barrier in the study by Goodwin et al.[22]. Participants noted that the preparation of the drug (drawn up from two vials for an adult dose) could be a barrier in a time pressured situation.

Age was also found to influence TXA administration with younger patients generally more likely to receive TXA[18,19,24,26,29], although the difference in age was often of questionable clinical significance. However, the evidence around a sex difference in administration was very clear[31]. Female patients are less likely to be treated with TXA in all risk categories despite TXA reducing trauma deaths to a similar extent in men and women. The disparity was greatest in older women and women at lower risk.

Following the 2019 CRASH3 study and subsequent sub analyses[3], TXA has been indicated in the UK for adult patients with isolated traumatic head injury (CGS score of 12 or less) since July 2020[37]. However, all of the studies in this review used data predominantly predating the reporting of CRASH3. The Nutbeam et al. data did briefly overlap the CRASH3 publication, but it did not specifically report on head injuries[31]. The two van Wessem papers also briefly overlapped but they specifically excluded patients with isolated head injuries[18,19]. Of the three papers looking at trauma patients with severe brain injury all three showed patient age and the severity of injury to be

For peer review only

influencing factors, with more severely injured patients more likely to receive TXA[17–19]. However, the influence of age on this patient group was mixed and requires further study. It seems there is a lack of evidence around the factors influencing TXA administration in patients with severe head injuries and in the case of isolated head injury this may be due to the relatively recent changes to protocols around TXA administration for these patients.

Other important barriers to administration identified by Goodwin et al. included a lack of knowledge and experience with TXA, a lack of resources and difficulty in identifying patients at risk of bleeding[22]. These findings may help to explain the low protocol compliance identified in this review, although none of the studies examined causal factors in detail. Injury severity played a role in administration, and although patients who are more severely injured are more likely to receive TXA[17,24,26,38], the measure used to assess injury severity in the included studies was often the ISS, which can only be calculated accurately following hospital assessment. Therefore, it is unclear what measure of severity pre-hospital clinicians are using to determine the need for TXA administration, and this may introduce additional variability.

A new scoring system to identify trauma patients at risk of bleeding has recently been published. This showed that the proportion of patients receiving pre-hospital TXA could be increased through the use of the Bleeding Audit Triage Trauma (BATT) score[8]. The BATT score is calculated using a weighted system including age, systolic blood pressure, GCS, respiratory rate, heart rate and whether the injury was penetrating or involved high velocity trauma. This adds weight to the idea that clinical observations are commonly used, and potentially effective, in guiding more accurate pre-hospital TXA administration. A challenge that emerges is how to help pre-hospital clinicians identify and treat not just patients at highest risk of bleeding following trauma, but also those at lower risk who may nevertheless benefit from TXA administration, given that the drug can be safely administered to a wide spectrum of patients with traumatic bleeding, and should not be restricted to the most severely injured[39]. The newly validated pre-hospital BATT score shows promise in helping to identify these lower risk patients[8]. Treating patients with a BATT score of just 2 or more (out of 27) would result in 26 fewer deaths per 10,000 trauma patients.

CONCLUSIONS

This review highlights a lack of high-quality research addressing the factors that influence pre-hospital TXA administration, particularly in children or patients with isolated head injuries. Common factors identified in this review suggest a host of system and individual-level factors that may be important in determining whether TXA is administered to trauma patients in the pre-hospital setting. These include: knowledge and skills; consequences and social influences; injury type (including severity, injury site and mechanism of injury); protocols; resources; priorities; patient age; patient sex. Despite the well-established benefits of TXA in trauma, and the central role of time to administration[4,5], pre-hospital administration rates remain low[9,26]. In order to address this evidence-practice gap it is essential that the factors influencing pre-hospital administration are better understood.

ABBREVIATIONS

AIS: Abbreviated Injury Scale

BATT: Bleeding Audit Triage Trauma

EMS: Emergency Medical Services

For peer review only

GCS: Glasgow Coma Score

HEMS: Helicopter Emergency Medical Services

ICTRP: International Clinical Trials Registry Platform

IDF: Israeli Defence Force

ISS: Injury Severity Score

EPHPP: Effective Public Health Practice Project

TARN: Trauma Audit and Research Network

TXA: Tranexamic Acid

UK: United Kingdom

DECLARATIONS

Ethics approval and consent to participate

Not applicable

Consent for publication

Not applicable

Availability of data and materials

The datasets generated and analysed during the review are not publicly available but are available from the corresponding author on reasonable request.

Competing interests

The authors declare that they have no competing interests.

Funding

This review was supported by Research Capability Funding from the South Western Ambulance Service NHS Foundation Trust and the National Institute for Health Research Applied Research Collaboration South West Peninsula. The views expressed in this publication are those of the authors and not necessarily those of the National Institute for Health Research of the Department of Health and Social Care.

Author contributions

HN participated in review conception, design and coordination, searches, paper selection, quality assessment, data extraction, data synthesis and drafted the manuscript. NK participated in searches, paper selection, quality assessment and data extraction. KK participated in conception, design, paper selection, quality assessment and data extraction. SV participated in conception, design and coordination and paper selection. JB, MR, AB, LG and HT participated in conception and design. JTC participated in design and data synthesis. SB participated in design and undertook database searches. All authors were responsible for critical revision of the manuscript for publication and approved the final version to be published.

Acknowledgements

For peer review only

We would like to thank Professor Ian Roberts (London School of Hygiene & Tropical Medicine), Professor Tim Coats (University of Leicester) and Dr Francois-Xavier Ageron (University of Lausanne) for their assistance during this project.

Registration
PROSPERO registration number CRD42020162943.

REFERENCES

1 National Audit Office. Major Trauma care in England. 2010.

2 Olldashi F, Kerçi M, Zhurda T, *et al.* Effects of tranexamic acid on death, vascular occlusive events, and blood transfusion in trauma patients with significant haemorrhage (CRASH-2): A randomised, placebo-controlled trial. *The Lancet* 2010;**376**:23–32. doi:10.1016/S0140-6736(10)60835-5

3 Roberts I, Shakur-Still H, Aeron-Thomas A, *et al.* Effects of tranexamic acid on death, disability, vascular occlusive events and other morbidities in patients with acute traumatic brain injury (CRASH-3): A randomised, placebo-controlled trial. *The Lancet* 2019;**394**:1713–23. doi:10.1016/S0140-6736(19)32233-0

4 Roberts I, Edwards P, Prieto D, *et al.* Tranexamic acid in bleeding trauma patients: An exploration of benefits and harms. *Trials* 2017;**18**:48. doi:10.1186/s13063-016-1750-1

5 Olldashi F, Kerçi M, Zhurda T, *et al.* The importance of early treatment with tranexamic acid in bleeding trauma patients: An exploratory analysis of the CRASH-2 randomised controlled trial. *The Lancet* 2011;**377**:1101.e1-1101.e2. doi:10.1016/S0140-6736(11)60278-X

6 Gayet-Ageron A, Prieto-Merino D, Ker K, *et al.* Effect of treatment delay on the effectiveness and safety of antifibrinolytics in acute severe haemorrhage: a meta-analysis of individual patient-level data from 40 138 bleeding patients. *The Lancet* 2018;**391**:125–32. doi:10.1016/S0140-6736(17)32455-8

7 Paudyal P, Smith J, Robinson M, *et al.* Tranexamic acid in major trauma: implementation and evaluation across South West England. *Eur J Emerg Med* 2017;**24**:44–8.

8 Ageron FX, Coats TJ, Darioli V, *et al.* Validation of the BATT score for prehospital risk stratification of traumatic haemorrhagic death: usefulness for tranexamic acid treatment criteria. *Scand J Trauma Resusc Emerg Med* 2021;**29**:6. doi:10.1186/s13049-020-00827-5

9 Marsden MER, Rossetto A, Duffield CAB, *et al.* Prehospital tranexamic acid shortens the interval to administration by half in Major Trauma Networks: a service evaluation. *Emergency Medicine Journal* 2019;**36**:395–400. doi:10.1136/emermed-2018-208118

10 Coats TJ, Fragoso-Iñiguez M, Roberts I. Implementation of tranexamic acid for bleeding trauma patients: a longitudinal and cross-sectional study. *Emergency Medicine Journal* 2019;**36**:78–81. doi:10.1136/emermed-2018-207693

11 Olaussen A, Semple W, Oteir A, *et al.* Paramedic literature search filters: Optimised for clinicians and academics. *BMC Med Inform Decis Mak* 2017;**17**:146. doi:10.1186/s12911-017-0544-z

For peer review only

12 Quality Assessment Tool for Quantitative Studies. https://www.ephpp.ca/quality-assessment-tool-for-quantitative-studies/ (accessed 27 Jan 2021).

13 Armijo-Olivo S, Stiles CR, Hagen NA, *et al.* Assessment of study quality for systematic reviews: A comparison of the Cochrane Collaboration Risk of Bias Tool and the Effective Public Health Practice Project Quality Assessment Tool: Methodological research. *J Eval Clin Pract* 2012;**18**:12–8. doi:10.1111/j.1365-2753.2010.01516.x

14 CASP CHECKLISTS - CASP - Critical Appraisal Skills Programme. https://casp-uk.net/casp-tools-checklists/ (accessed 27 Jan 2021).

15 Greenfield K, Holley S, Schoth DE, *et al.* Barriers and facilitators experienced by patients, carers and healthcare professionals when managing symptoms in infants, children and young people at end-of-life: a mixed methods systematic review protocol. doi:10.1136/bmjopen-2019-030566

16 Neeki MM, Dong F, Toy J, *et al.* Tranexamic acid in civilian trauma care in the California Prehospital Antifibrinolytic Therapy study. *Western Journal of Emergency Medicine* 2018;**19**:977–86. doi:10.5811/westjem.2018.8.39336

17 Bossers SM, Loer SA, Bloemers FW, *et al.* Association between Prehospital Tranexamic Acid Administration and Outcomes of Severe Traumatic Brain Injury. *JAMA Neurol* 2021;**78**:328–45. doi:10.1001/jamaneurol.2020.4596

18 van Wessem KJP, Jochems D, Leenen LPH. The effect of prehospital tranexamic acid on outcome in polytrauma patients with associated severe brain injury. *European Journal of Trauma and Emergency Surgery* 2022;**48**:1589–99. doi:10.1007/s00068-021-01827-5

19 van Wessem KJP, Luke •, Leenen PH. Does Liberal Prehospital and In-Hospital Tranexamic Acid Influence Outcome in Severely Injured Patients? A Prospective Cohort Study. *World J Surg* 2021;**45**:2398–407. doi:10.1007/s00268

20 El-Menyar A, Sathian B, Wahlen BM, *et al.* Prehospital administration of tranexamic acid in trauma patients: A 1:1 matched comparative study from a level 1 trauma center. *American Journal of Emergency Medicine* 2020;**38**:266–71. doi:10.1016/j.ajem.2019.04.051

21 Vu EN, Schlamp RS, Wand RT, *et al.* Prehospital Use of Tranexamic Acid for Hemorrhagic Shock in Primary and Secondary Air Medical Evacuation. *Air Med J* 2013;**32**:289–92. doi:10.1016/j.amj.2013.05.001

22 Goodwin L, Nicholson H, Robinson M, *et al.* Barriers and facilitators to the administration of prehospital tranexamic acid: a paramedic interview study using the theoretical domains framework. *Emerg Med J* 2022;**39**:540–6. doi:10.1136/emermed-2020-210622

23 McQueen C, Wyse M. The introduction of Tranexamic Acid to Helicopter Emergency Medical Service operations in a regional service in the United Kingdom. *Trauma (United Kingdom)* 2013;**15** **(4)**:344.https://ovidsp.ovid.com/ovidweb.cgi?T=JS&CSC=Y&NEWS=N&PAGE=fulltext&D=emed14&AN=71418011

24 Wong D, Su G, Mabasa VH, *et al.* Assessing the clinical utilization of tranexamic acid by paramedics for patients with major trauma (ACUTE). *Canadian Journal of Emergency Medicine* 2021;**23**:219–22. doi:10.1007/s43678-020-00040-4

For peer review only

25 Benov A, Shkolnik I, Glassberg E, *et al.* Prehospital Trauma Experience of the Israel Defense Forces on the Syrian Border 2013-2017. *J Trauma Acute Care Surg* Published Online First: 2019. doi:10.1097/TA.0000000000002217

26 Fisher AD, Carius BM, April MD, *et al.* An Analysis of Adherence to Tactical Combat Casualty Care Guidelines for the Administration of Tranexamic Acid. *Journal of Emergency Medicine* 2019;**57**:646–52. doi:10.1016/j.jemermed.2019.08.027

27 Ng M, Perrott J, Burgess S. Evaluation of tranexamic acid in trauma patients: A retrospective quantitative analysis. *American Journal of Emergency Medicine* 2019;**37**:444–9. doi:10.1016/j.ajem.2018.06.010

28 Tsur N, Benov A, Nadler R, *et al.* Neck injuries - israel defense forces 20 years' experience. *Injury* Published Online First: 2020. doi:https://dx.doi.org/10.1016/j.injury.2020.09.042

29 Wafaisade A, Lefering R, Bouillon B, *et al.* Prehospital administration of tranexamic acid in trauma patients. *Crit Care* 2016;**20**:1–9. doi:10.1186/s13054-016-1322-5

30 Betelman Mahalo Y, Avital G, Radomislensky I, *et al.* Pain management for casualties receiving lifesaving interventions in the prehospital scenario: Raising awareness of our human nature. *J Trauma Acute Care Surg* 2021;**91**:S201–5. doi:10.1097/TA.0000000000003295

31 Nutbeam T, Roberts I, Weekes L, *et al.* Use of tranexamic acid in major trauma: a sex-disaggregated analysis of the Clinical Randomisation of an Antifibrinolytic in Significant Haemorrhage (CRASH-2 and CRASH-3) trials and UK trauma registry (Trauma and Audit Research Network) data. *Br J Anaesth* 2022;**129**:191–9. doi:10.1016/j.bja.2022.03.032

32 Nadler R, Gendler S, Benov A, *et al.* Tranexamic acid at the point of injury: The Israeli combined civilian and military experience. *Journal of Trauma and Acute Care Surgery* 2014;**77**:S146–50. doi:10.1097/ta.0000000000000325

33 Lipsky AM, Abramovich A, Nadler R, *et al.* Tranexamic acid in the prehospital setting: Israel Defense Forces' initial experience. *Injury* 2014;**45**:66–70. doi:10.1016/j.injury.2013.08.025

34 Nadler R, Tsur AM, Lipsky AM, *et al.* Trends in combat casualty care following the publication of clinical practice guidelines. *J Trauma Acute Care Surg* 2021;**91**:S194–200. doi:10.1097/TA.0000000000003280

35 Schauer SG, April MD, Naylor JF, *et al.* QuikClot® Combat Gauze® Use by Ground Forces in Afghanistan The Prehospital Trauma Registry Experience. *J Spec Oper Med* 2016;**17**:101–6.

36 Godfrey BW, Martin A, Chestovich PJ, *et al.* Patients with multiple traumatic amputations: An analysis of operation enduring freedom joint theatre trauma registry data. *Injury* 2017;**48**:75–9. doi:10.1016/j.injury.2016.08.008

37 Head Injury Algorithm - aace.org.uk. https://aace.org.uk/jrcalc-updates-2019/head-injury-algorithm/ (accessed 24 Jan 2023).

38 Morrison JJ, Dubose JJ, Rasmussen TE, *et al.* Military application of tranexamic acid in trauma emergency resuscitation (MATTERs) study. *Archives of Surgery* 2012;**147**:113–9. doi:10.1001/archsurg.2011.287

For peer review only

39 Roberts I, Perel P, Prieto-Merino D, *et al.* Effect of tranexamic acid on mortality in patients with traumatic bleeding: Prespecified analysis of data from randomised controlled trial. *BMJ (Online)* 2012;**345**. doi:10.1136/bmj.e5839

For peer review only

APPENDIX 1- Literature search report

Bibliographic database searches

Database: AMED

Host: EBSCO

Data Parameters: n/a

Date Searched: 10/1/2020

Searcher: SB

Hits: 2

Strategy:

1. TI "tranexamic acid" OR AB "tranexamic acid"
2. TI TXA OR AB TXA
3. TI cyklokapron OR AB cyklokapron
4. 1 or 2 or 3

Notes: Date limited 2010 – to date

Database: CENTRAL

Host: Cochrane Library

Data Parameters: Issue 1 of 12, January 2020

Date Searched: 10/10/2020

Searcher: SB

Hits: 196

Strategy:

#1 ("tranexamic acid"):ti OR ("tranexamic acid"):ab

#2 (TXA):ti OR (TXA):ab

#3 (cyklokapron):ti OR (cyklokapron):ab

#4 MeSH descriptor: [Tranexamic Acid] this term only

#5 #1 or #2 or #3 or #4

#6 (trauma*):ti OR (trauma*):ab

#7 (paramedic* or ems or emt or prehospital or "pre hospital" or "first responder*" or "emergency medical technician*" or "emergency services" or Ambulance* or HEMS or "field triage" or "out-of-hospital"):ti OR (paramedic* or ems or emt or prehospital or "pre hospital" or "first

For peer review only

responder*" or "emergency medical technician*" or "emergency services" or Ambulance* or HEMS or "field triage" or "out-of-hospital"):ab

#8 MeSH descriptor: [Ambulances] this term only

#9 MeSH descriptor: [Emergency Medical Technicians] this term only

#10 MeSH descriptor: [Air Ambulances] this term only

#11 MeSH descriptor: [Emergency Medical Services] this term only

#12 #6 or #7 or #8 or #9 or #10 or #11

#13 #5 and #12

Notes: Date limited 2010 – 2020

Database: CINAHL
Host: EBSCO
Data Parameters: n/a
Date Searched: 13/1/2020
Searcher: SB
Hits: 313
Strategy:

1. TI "tranexamic acid" OR AB "tranexamic acid"
2. TI TXA OR AB TXA
3. TI cyklokapron OR AB cyklokapron
4. (MH "Tranexamic Acid")
5. TI trauma* OR AB trauma*
6. TI ((paramedic* or ems or emt or prehospital or "pre hospital" or "first responder*" or "emergency medical technician*" or "emergency services" or Ambulance* or HEMS or "field triage" or "out-of-hospital")) OR AB ((paramedic* or ems or emt or prehospital or "pre hospital" or "first responder*" or "emergency medical technician*" or "emergency services" or Ambulance* or HEMS or "field triage" or "out-of-hospital"))
7. (MH "Ambulances")
8. (MH "Aeromedical Transport")
9. (MH "Emergency Medical Services+")
10. S1 OR S2 OR S3 OR S4
11. S5 OR S6 OR S7 OR S8 OR S9
12. S10 AND S11

For peer review only

Notes: Date limited 2010 – to date; English language results only

Database: Cochrane Database of Systematic Reviews (CDSR)

Host: Cochrane Library

Data Parameters: Issue 1 of 12, January 2020

Date Searched: 10/10/2010

Searcher: SB

Hits: 6

Strategy: see CENTRAL search

Notes: Date limited 2010 – 2020

Database: Conference Proceedings Citation Index – Science (CPCI-S)

Host: Clarivate Analytics

Data Parameters: n/a

Date Searched: 10/1/2020

Searcher: SB

Hits: 86

Strategy:

1. TOPIC: ("tranexamic acid")
2. TOPIC: (TXA)
3. TOPIC: (cyklokapron)
4. #1 OR #2 OR #3
5. TOPIC: (trauma*)
6. TOPIC: (paramedic* or ems or emt or prehospital or "pre hospital" or "first responder*" or "emergency medical technician*" or "emergency services" or Ambulance* or HEMS or "field triage" or "out-of-hospital")
7. #5 OR #6
8. (#7 AND #4) AND LANGUAGE: (English) Indexes=CPCI-S Timespan=2010-2020

Database: Embase

Host: Ovid

Data Parameters: 1974 to 2020 January 09

Date Searched: 10/1/2020

Searcher: SB

Hits: 1250

Strategy:

For peer review only

1. "tranexamic acid".tw.
2. TXA.tw.
3. cyklokapron.tw.
4. tranexamic acid/
5. or/1-4
6. trauma*.tw.
7. (paramedic* or ems or emt or prehospital or "pre hospital" or "first responder*" or "emergency medical technician*" or "emergency services" or Ambulance* or HEMS or "field triage" or "out-of-hospital").tw.
8. exp ambulance/
9. rescue personnel/
10. air medical transport/
11. exp emergency health service/
12. or/6-11
13. 5 and 12
14. limit 13 to (english language and yr="2010 –Current")

Database: MEDLINE ALL

Host: Ovid

Data Parameters: 1946 to January 09, 2020

Date Searched: 10/1/2020

Searcher: SB

Hits: 513

Strategy:

1. "tranexamic acid".tw.
2. TXA.tw.
3. cyklokapron.tw.
4. Tranexamic Acid/
5. or/1-4
6. trauma*.tw.
7. (paramedic* or ems or emt or prehospital or "pre hospital" or "first responder*" or "emergency medical technician*" or "emergency services" or Ambulance* or HEMS or "field triage" or "out-of-hospital").tw.
8. Ambulances/
9. Emergency Medical Technicians/
10. Air Ambulances/

For peer review only

11. Emergency Medical Services/
12. or/6-11
13. 5 and 12
14. Limit 13 to (english language and yr="2010 –Current")

Table A1. Total number of hits per database and unique records

Database	Hits
AMED	2
CENTRAL	196
CINAHL	313
CDSR	6
CPCI-S	86
Embase	1250
MEDLINE ALL	513
**Total records**	**2366**
**Duplicate records**	**605**
**Unique records**	**1761**

OpenGrey search

URL: http://www.opengrey.eu/

Date Searched: 09/03/2021

Searcher: HN

Hits: 11

Strategy: "tranexamic acid" OR TXA

Clinical trials registry search

Registry: ClinicalTrials.gov

URL: https://clinicaltrials.gov/

Date Searched: 24/06/2020

Searcher: HN

Hits: 27

Strategy:

Intervention/treatment: "tranexamic acid" OR TXA OR cyklokapron

Title/Acronym: trauma OR prehospital OR "pre-hospital" OR emergency

For peer review only

Update searches

Bibliographic database searches

Database: AMED

Host: EBSCO

Data Parameters: n/a

Date Searched: 14/12/2020

Searcher: SB

Hits: 0

Strategy: see above (date limited 2020 to date of search)

Database: CENTRAL

Host: Cochrane Library

Data Parameters: Issue 12 of 12, December 2020

Date Searched: 14/12/2020

Searcher: SB

Hits: 65

Strategy: see above (date limited 2020 to date of search)

Database: CINAHL

Host: EBSCO

Data Parameters: n/a

Date Searched: 14/12/2020

Searcher: SB

Hits: 68

Strategy: see above (date limited 2020 to date of search)

Database: Cochrane Database of Systematic Reviews (CDSR)

Host: Cochrane Library

Data Parameters: Issue 12 of 12, December 2020

Date Searched: 14/12/2020

Searcher: SB

Hits: 0

Strategy: see above (date limited 2020 to date of search)

For peer review only

Database: Conference Proceedings Citation Index – Science (CPCI-S)

Host: Clarivate Analytics

Data Parameters: n/a

Date Searched: 14/12/2020

Searcher: SB

Hits: 7

Strategy: see above (date limited 2020 to date of search)

Database: Embase

Host: Ovid

Data Parameters: 1974 to 2020 December 11

Date Searched: 14/12/2020

Searcher: SB

Hits: 227

Strategy: see above (date limited 2020 to date of search)

Database: MEDLINE ALL

Host: Ovid

Data Parameters: 1946 to December 11, 2020

Date Searched: 14/12/2020

Searcher: SB

Hits: 132

Strategy: see above (date limited 2020 to date of search)

Table A2. Total number of hits per database and unique records

**Database**	**Hits**
AMED	0
CENTRAL	65
CINAHL	68
CDSR	0
CPCI-S	7
Embase	227
MEDLINE ALL	132
**Total records**	**499**
**Duplicate records**	**189**
**Unique records**	**310**

For peer review only

Clinical trials registry search

Registry: ClinicalTrials.gov

URL: https://clinicaltrials.gov/

Date Searched: 25/01/2021

Searcher: HN

Hits: 30

Strategy: see above

Update searches 2

Bibliographic database searches

Database: AMED

Host: EBSCO

Data Parameters: n/a

Date Searched: 8/6/2022

Searcher: SB

Hits: 4

Strategy: see above (date limited 2020 to date of search)

Database: CENTRAL

Host: Cochrane Library

Data Parameters: Issue 5 of 12, May 2022

Date Searched: 8/6/2022

Searcher: SB

Hits: 132

Strategy: see above (date limited 2020 to date of search)

Database: CINAHL

Host: EBSCO

Data Parameters: n/a

Date Searched: 9/6/2022

Searcher: SB

Hits: 712

Strategy: see above (date limited 2020 to date of search)

Database: Cochrane Database of Systematic Reviews (CDSR)

For peer review only

Host: Cochrane Library

Data Parameters: Issue 6 of 12, June 2022

Date Searched: 8/6/2022

Searcher: SB

Hits: 0

Strategy: see above (date limited 2020 to date of search)

Database: Conference Proceedings Citation Index – Science (CPCI-S)

Host: Clarivate Analytics

Data Parameters: n/a

Date Searched: 8/6/2022

Searcher: SB

Hits: 15

Strategy: see above (date limited 2020 to date of search)

Database: Embase

Host: Ovid

Data Parameters: 1974 to 2022 June 07

Date Searched: 8/6/2022

Searcher: SB

Hits: 571

Strategy: see above (date limited 2020 to date of search)

Database: MEDLINE ALL

Host: Ovid

Data Parameters: 1946 to June 07, 2022

Date Searched: 8/6/2022

Searcher: SB

Hits: 306

Strategy: see above (date limited 2020 to date of search)

Table A3. Total number of hits per database and unique records

Database	Hits
AMED	4
CENTRAL	132
CINAHL	712

For peer review only

CDSR	0
CPCI-S	15
Embase	571
MEDLINE ALL	306
**Total records**	**1740**
**Duplicate records**	**473**
**Duplicate record with previous search**	**222**
**Unique records**	**1045**

OpenGrey search

URL: http://www.opengrey.eu/

Date Searched: 25/01/2021

Searcher: HN

Hits: 18

Strategy: "tranexamic acid" OR TXA

Clinical trials registry search

Registry: ClinicalTrials.gov

URL: https://clinicaltrials.gov/

Date Searched: 25/01/2021

Searcher: HN

Hits: 33

Strategy: see above

For peer review only

APPENDIX 2 - Inclusion and exclusion criteria

PEOS categories	Inclusion criteria	Exclusion criteria
**Population**	Any patients who have experienced traumatic injury	Patients receiving TXA who have not experienced traumatic injury or who have superficial minor wounds.<br><br>Patients experiencing obstetric, post-partum haemorrhage.
**Exposure**	Factors influencing the decision to administer TXA.	
**Outcome**	Papers detailing the characteristics of patients who did or did not receive TXA in the pre-hospital setting.<br><br>Papers detailing characteristics of pre-hospital clinicians who administered TXA.	TXA administration in hospital. Studies that solely report the clinical and/or cost effectiveness of TXA, dose or route of administration rather than the factors that influenced administration will be excluded.
**Setting**	Any pre-hospital setting where patients are treated by pre-hospital clinicians outside of a hospital or primary care environment.	Any hospital or primary care setting.
**Study design**	Any primary research.	Review articles, editorials and letters.

For peer review only

APPENDIX 3 – Variable sought during data extraction

List of variables for which data was sought during data extraction:

- Citation
- Country
- Study aims
- Patient and clinician population characteristics
- Primary outcome
- Inclusion and exclusion criteria
- Recruitment method
- Study Design
- Sample size and sampling method
- Data source
- Intervention and comparator group (if applicable)
- Date and duration of data collection
- Setting
- Analysis
- Factors influencing TXA administration

For peer review only

APPENDIX 4 – PRISMA diagram

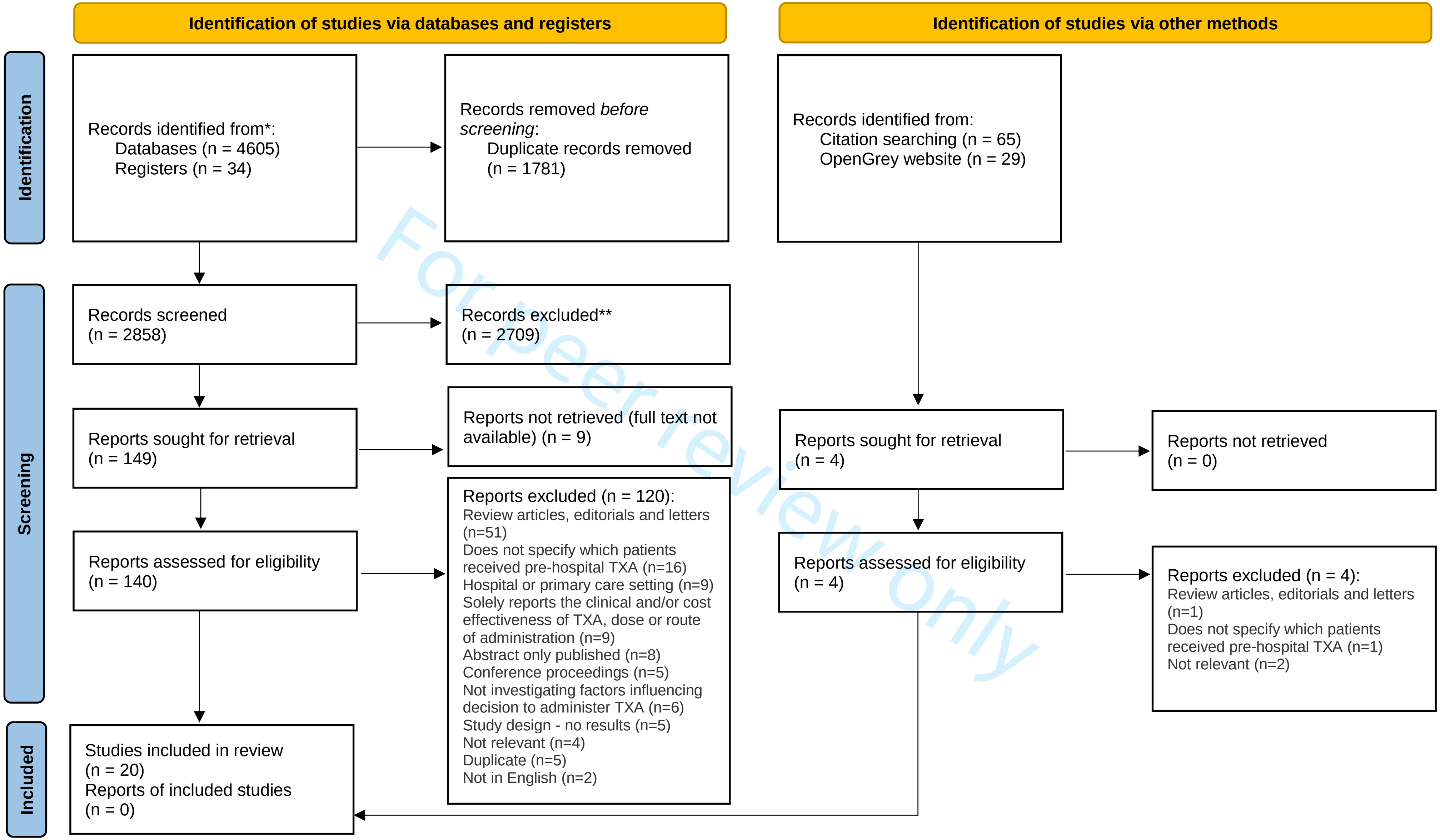

*Consider, if feasible to do so, reporting the number of records identified from each database or register searched (rather than the total number across all databases/registers).
**If automation tools were used, indicate how many records were excluded by a human and how many were excluded by automation tools.

From: Page MJ, McKenzie JE, Bossuyt PM, Boutron I, Hoffmann TC, Mulrow CD, et al. The PRISMA 2020 statement: an updated guideline for reporting systematic reviews. BMJ 2021;372:n71. doi: 10.1136/bmj.n71. For more information, visit: http://www.prisma-statement.org/

For peer review only - http://bmjopen.bmj.com/site/about/guidelines.xhtml

APPENDIX 5- Characteristics of included civilian studies (n=13).

First Author (date), country	Study design	Patient number	Data source	Dates and duration of data collection	Population	Factors influencing TXA administration
Bossers (2021), Netherlands	Observational mulitcentre cohort study	1827	Brain Injury: Prehospital Registry of Outcome, Treatments and Epidemiology of Cerebral Trauma (BRAINPROTECT) study database.	February 2012- December 2017, 5 years 10 months	Adult patients with severe TBI attended by HEMS	**Patient age** - patients receiving TXA older than those not receiving TXA (47 vs 45 years).<br><br>**Injury type, severity -** patients receiving TXA had higher Injury Severity Score (ISS) (27 vs 26), lower prehospital Glasgow Coma Score (GCS) (4 vs 5) and higher heart rate (98 vs 92).
El-Menyar (2020), Qatar	Retrospective observational, case control	204	Qatar National Trauma Registry	January 1 2017 to September 30 2018; 1 year 9 months	Adult trauma patients	**Resources** – patients did not receive TXA if critical care paramedics unavailable.
Goodwin (2021), UK	Qualitative interview study	18	UK paramedic interviews	2019	N/A	**Knowledge and skills** - inadequate training and a lack of knowledge of the effects of TXA or the evidence base behind its use and a lack of exposure to trauma patients were barriers to its administration.<br>**Resources -** a lack of time and staffing was a barrier to administration. Helicopter Emergency Medicine Services (HEMS) attendance was a barrier to some as they preferred to wait for a HEMS team member to administer it.<br>**Protocol -** guidelines felt restrictive or confusing. Disparity between paramedic, HEMS and doctor TXA protocols causes confusion. The drug preparation and administration route were seen as barriers to its use.<br>**Consequences and social influences-** the benefits of TXA were seen to outweigh the risks. TXA was seen to signal a major trauma patient. Fear of repercussion for administering TXA inappropriately. Opinion of colleagues seen to influence TXA administration.

For peer review only

						**Priorities** - TXA not seen as a priority, with administering fluids or distracting injuries often taking precedent. Three-hour window of administration may reduce the perceived urgency of administration. The stress associated with trauma jobs may lead to TXA being overlooked or deprioritised. **Injury type, severity - r**isk of bleeding influenced administration including clinical observations and patient presentation. Participants found identifying patients at risk of bleeding difficult. **Injury type, Mechanism of Injury (MOI) -** uncertainty over which injuries/conditions TXA is indicated for. MOI and type of injury were part of identify patients at risk of bleeding. **Injury type, site** - less obvious bleeding including occult/internal haemorrhage was harder to identify.
Marsden (2019), UK	Retrospective service evaluation	661	TARN, local trauma registries and individual patient hospital records	January 1 2017 to December 31 2017; 1 year	Adult and paediatric trauma patients	**Injury type, MOI** – road traffic collisions more likely to receive TXA, falls less likely. **Injury type, severity** – patients given TXA more likely to have heart rate and blood pressure suggestive of bleeding. **Resources** – patients given TXA were more likely to be treated by a physician-led crew.
McQueen (2013), UK	Retrospective service evaluation	123	HEMS clinical database.	6 months - Dates not stated.	Major trauma patients attended by HEMS	**Protocol** - clinician judgement used to guide administration outside of the protocol. **Injury type, MOI** – most TXA patients had multiple injuries. **Resources** - time constraints on scene and the absence of a doctor as part of the HEMS crew meant TXA not given.
Neeki (2018), USA	Observational cohort study	724	Electronic medical record and trauma registry	March 2015 to July 2017; 2 years 4 months	Adult trauma patients	**Resources** - not all Emergency Medicine Service providers carrying TXA.
Ng (2018), Canada	Retrospective observational	117	British Columbia Trauma Registry and Royal Columbian Hospital records.	April 2012 to June 2015; 3 years 2 months.	Adult trauma patients	**Injury type, severity** – more patients receiving TXA had moderate (9-12) or severe (3-8) GCS scores than those not receiving TXA.
Nutbeam (2022), UK	Retrospective observational	216,364	Trauma and Audit Research	January 1 2017 to Dec		**Sex** - female patients less likely to receive TXA (OR 0.35, 95% CI, 0.33-0.36).

For peer review only - http://bmjopen.bmj.com/site/about/guidelines.xhtml

			Network (TARN) registry	31 2020; 4 years	Adult trauma patients	**Injury type, severity** - females less likely than males to receive TXA in all ISS categories.<br>**Injury type, MOI** - females less likely than males to receive TXA for all injury mechanisms except motor vehicle crashes.
van Wessem (2022), Netherlands	Prospective cohort study	234	Hospital data	November 2013; 7.5 years	Adult trauma patients with associated severe TBI	**Patient age** - patients receiving TXA younger than those not receiving TXA (42 vs 53 years).<br>**Injury type, severity -** patients needing pre-hospital intubation, urgent laparotomy or with more deranged physiology more likely to have received TXA.
van Wessem (2021), Netherlands	Population based Prospective cohort study	422	Hospital data	November 2013; 7 years	Adult trauma patients with associated severe TBI	**Patient age** - patients receiving TXA younger than those not receiving TXA (41 vs 51 years).<br>**Injury type, severity -** patients receiving TXA slightly more severely injured, had higher Abbreviated Injury Scale (AIS) Head scores and more often prehospitally intubated.
Vu (2013), USA	Case series	13	British Columbia Ambulance Service AirEvac and Critical Care Operation data system.	4 months - Dates unclear	Adult major trauma patients	**Priorities** - balancing critical interventions, resuscitation and short flight times meant some eligible patients did not receive TXA.
Wafaisade (2016), Germany	Retrospective observational	5765	ADAC Air Rescue Service prehospital database and German Trauma Society Trauma registry	January 1 2012 to December 31 2014; 3 years	Critically injured adult trauma patients	**Injury type, severity** - patients needing pre-hospital intubation or chest tube placement more likely to receive TXA.<br>**Injury type, site** - patients with AIS ≥3 for abdomen or extremities more likely to receive TXA. Patients with AIS ≥3 for head or thorax less likely to receive TXA.<br>**Age** - patients over 60 years old less likely to receive TXA.
Wong (2021), Canada	Retrospective observational	100	Hospital charts and British Columbia Trauma Registry	April 1 2016 to March 31 2017; 1 year	Adult trauma patients	**Age** - TXA group notably younger (38.2 years vs 49.1 years)<br>**Injury type, severity** - TXA group had higher mean ISS and more patients with hypotension.<br>**Resources** - more patients receiving TXA had a paramedic of higher certification level in attendance than those who did not receive TXA.

For peer review only

APPENDIX 6- Characteristics of included military setting studies (n=7).

First Author (date), country	Study design	Patient number	Data source	Dates and duration of data collection	Population	Factors influencing TXA administration
Benov (2019), Israel-Syrian Border	Retrospective observational	2339	Israel Defences Forces Trauma Registry	February 12 2013 to December 31 2017; 4 Years 10 months 19 days	Combat injuries, Syrian adult and paediatric refugees	**Protocol** – TXA for penetrating torso injury regardless of haemodynamic status. All patients receiving freeze-dried plasma with a known time of injury <3 hours received TXA.<br>**Injury type, MOI** – majority of TXA patients had penetrating injury.
Fisher (2019), Iraq and Afghanistan	Retrospective observational	28,222	The Department of Defence Trauma Registry.	January 2007 to August 2016; 9 years and 8 months.	Adult trauma patients	**Injury type, severity** – patients with higher ISS, tourniquet application or serious injuries to the thorax, abdomen, extremities and skin more likely to receive TXA.<br>**Injury type, MOI** - explosive injury and gunshot wounds were more likely to receive TXA. More likely to have explosive injuries and less likely gunshot wounds in TXA patients who had a tourniquet applied.<br>**Patient age** - amputation patients receiving TXA were younger than those not receiving TXA (22 vs 25 years).
Lipsky (2014), Israel	Retrospective observational	40	Israel Defences Forces Trauma Registry	December 2011 to February 2013; 1 year 2 months.	Adult trauma patients	**Protocol** – 30% of TXA administrations had no clear indication for TXA administration. Altered level of consciousness mistakenly categorised as a sign of haemodynamic instability.<br>**Priorities** – some non-administrations due to tactical limitations, resuscitation or to avoid delaying evacuation.
Mahalo (2021), Israel	Retrospective observational	1059	Israel Defences Force Trauma Registry and Israel National Trauma Registry	2006 to 2017; 10 years	Adult civilian and military trauma patients	**Injury type, severity** - TXA administration associated with analgesic treatment.
Nadler (2014), Israel	Retrospective observational	94	Israel Defences Forces Trauma Registry and hospital charts	December 2011 to August 2013; 1 year 8 months	Adult civilian and military trauma patients	**Protocol** – more conservative protocol in the civilian service compared to the military service but higher proportion of patients given TXA outside of protocol (with clearance) by civilian service.

For peer review only

Nadler (2021), Israel	Retrospective observational	16529	Israel Defences Forces Trauma Registry and hospital charts	January 2006 to December 2018; 13 years	Adult civilian and military trauma patients	**Protocol -** New Clinical Practice Guidelines (indicating TXA at a heart rate of 130 instead of 110) introduced caused a significant decrease in the proportion of TXA administered. Only 22% of patients indicated for TXA received it.
Tsur (2020), Israel	Retrospective observational	3394	Israel Defences Forces Trauma Registry	January 1997 to December 2018; 11 years	Adult military trauma patients	**Injury type, site** – isolated neck injuries more likely to receive TXA than no-neck injuries.

For peer review only

PRISMA 2020 Checklist

Section and Topic	Item #	Checklist item	Location where item is reported
**TITLE**			
Title	1	Identify the report as a systematic review.	p.1
**ABSTRACT**			
Abstract	2	See the PRISMA 2020 for Abstracts checklist.	p.1-2
**INTRODUCTION**			
Rationale	3	Describe the rationale for the review in the context of existing knowledge.	p.2
Objectives	4	Provide an explicit statement of the objective(s) or question(s) the review addresses.	p.2
**METHODS**			
Eligibility criteria	5	Specify the inclusion and exclusion criteria for the review and how studies were grouped for the syntheses.	p.3. + Appendix 2
Information sources	6	Specify all databases, registers, websites, organisations, reference lists and other sources searched or consulted to identify studies. Specify the date when each source was last searched or consulted.	p.3 + Appendix 1
Search strategy	7	Present the full search strategies for all databases, registers and websites, including any filters and limits used.	Appendix 1
Selection process	8	Specify the methods used to decide whether a study met the inclusion criteria of the review, including how many reviewers screened each record and each report retrieved, whether they worked independently, and if applicable, details of automation tools used in the process.	p.3
Data collection process	9	Specify the methods used to collect data from reports, including how many reviewers collected data from each report, whether they worked independently, any processes for obtaining or confirming data from study investigators, and if applicable, details of automation tools used in the process.	p.3
Data items	10a	List and define all outcomes for which data were sought. Specify whether all results that were compatible with each outcome domain in each study were sought (e.g. for all measures, time points, analyses), and if not, the methods used to decide which results to collect.	p.3
	10b	List and define all other variables for which data were sought (e.g. participant and intervention characteristics, funding sources). Describe any assumptions made about any missing or unclear information.	p.3 + Appendix 3
Study risk of bias assessment	11	Specify the methods used to assess risk of bias in the included studies, including details of the tool(s) used, how many reviewers assessed each study and whether they worked independently, and if applicable, details of automation tools used in the process.	p.3
Effect measures	12	Specify for each outcome the effect measure(s) (e.g. risk ratio, mean difference) used in the synthesis or presentation of results.	N/A
Synthesis methods	13a	Describe the processes used to decide which studies were eligible for each synthesis (e.g. tabulating the study intervention characteristics and comparing against the planned groups for each synthesis (item #5)).	p.3
	13b	Describe any methods required to prepare the data for presentation or synthesis, such as handling of missing summary statistics, or data conversions.	N/A
	13c	Describe any methods used to tabulate or visually display results of individual studies and syntheses.	p.3
	13d	Describe any methods used to synthesize results and provide a rationale for the choice(s). If meta-analysis was performed, describe the model(s), method(s) to identify the presence and extent of statistical heterogeneity, and software package(s) used.	p.3
	13e	Describe any methods used to explore possible causes of heterogeneity among study results (e.g. subgroup analysis, meta-regression).	N/A
	13f	Describe any sensitivity analyses conducted to assess robustness of the synthesized results.	N/A
Reporting bias assessment	14	Describe any methods used to assess risk of bias due to missing results in a synthesis (arising from reporting biases).	p.3

For peer review only - http://bmjopen.bmj.com/site/about/guidelines.xhtml

PRISMA 2020 Checklist

Section and Topic	Item #	Checklist item	Location where item is reported
Certainty assessment	15	Describe any methods used to assess certainty (or confidence) in the body of evidence for an outcome.	p.3-4
**RESULTS**			
Study selection	16a	Describe the results of the search and selection process, from the number of records identified in the search to the number of studies included in the review, ideally using a flow diagram.	p.3-4 + Appendix 4
	16b	Cite studies that might appear to meet the inclusion criteria, but which were excluded, and explain why they were excluded.	N/A
Study characteristics	17	Cite each included study and present its characteristics.	Table 1 + Table 2 + Appendix 5 + Appendix 6
Risk of bias in studies	18	Present assessments of risk of bias for each included study.	p.3-4
Results of individual studies	19	For all outcomes, present, for each study: (a) summary statistics for each group (where appropriate) and (b) an effect estimate and its precision (e.g. confidence/credible interval), ideally using structured tables or plots.	Table 1 + Table 2 + Appendix 5 + Appendix 6
Results of syntheses	20a	For each synthesis, briefly summarise the characteristics and risk of bias among contributing studies.	p.4
	20b	Present results of all statistical syntheses conducted. If meta-analysis was done, present for each the summary estimate and its precision (e.g. confidence/credible interval) and measures of statistical heterogeneity. If comparing groups, describe the direction of the effect.	N/A
	20c	Present results of all investigations of possible causes of heterogeneity among study results.	p.3-10
	20d	Present results of all sensitivity analyses conducted to assess the robustness of the synthesized results.	N/A
Reporting biases	21	Present assessments of risk of bias due to missing results (arising from reporting biases) for each synthesis assessed.	p.3-4
Certainty of evidence	22	Present assessments of certainty (or confidence) in the body of evidence for each outcome assessed.	N/A
**DISCUSSION**			
Discussion	23a	Provide a general interpretation of the results in the context of other evidence.	p.10-12
	23b	Discuss any limitations of the evidence included in the review.	p.2, p.10-12
	23c	Discuss any limitations of the review processes used.	p.2, p.10-12
	23d	Discuss implications of the results for practice, policy, and future research.	p.10-12
**OTHER INFORMATION**			
Registration and protocol	24a	Provide registration information for the review, including register name and registration number, or state that the review was not registered.	p.2, p.14
	24b	Indicate where the review protocol can be accessed, or state that a protocol was not prepared.	p.2
	24c	Describe and explain any amendments to information provided at registration or in the protocol.	p.2
Support	25	Describe sources of financial or non-financial support for the review, and the role of the funders or sponsors in the review.	p.2, p.13

For peer review only

For peer review only - http://bmjopen.bmj.com/site/about/guidelines.xhtml

PRISMA 2020 Checklist

Section and Topic	Item #	Checklist item	Location where item is reported
Competing interests	26	Declare any competing interests of review authors.	p.13
Availability of data, code and other materials	27	Report which of the following are publicly available and where they can be found: template data collection forms; data extracted from included studies; data used for all analyses; analytic code; any other materials used in the review.	Available on request.

From: Page MJ, McKenzie JE, Bossuyt PM, Boutron I, Hoffmann TC, Mulrow CD, et al. The PRISMA 2020 statement: an updated guideline for reporting systematic reviews. BMJ 2021;372:n71. doi: 10.1136/bmj.n71

For more information, visit: http://www.prisma-statement.org/

For peer review only

Factors that influence the administration of tranexamic acid (TXA) to trauma patients in pre-hospital settings: A systematic review.

Journal:	*BMJ Open*
Manuscript ID	bmjopen-2023-073075.R1
Article Type:	Original research
Date Submitted by the Author:	10-May-2023
Complete List of Authors:	Nicholson, Helen; University of the West of England, College of Health, Science and Society<br>Scotney, Natalie; South Western Ambulance Service NHS Foundation Trust<br>Briscoe, Simon; University of Exeter, University of Exeter Medical School<br>Kirby, Kim; University of the West of England Department of Health and Social Sciences; South Western Ambulance Service NHS Foundation Trust, Research, Audit and Quality Improvement Department<br>Bedson, Adam; South Western Ambulance Service NHS Foundation Trust<br>Goodwin, Laura; University of the West of England, College of Health, Science and Society<br>Robinson , Maria; South Western Ambulance Service NHS Foundation Trust<br>Taylor, Hazel; University Hospitals Bristol NHS Foundation Trust, Research Design Service<br>Thompson Coon, Jo; University of Exeter, NIHR CLAHRC South West Peninsula<br>Voss, Sarah; University of the West of England, College of Health, Science and Society<br>Benger, Jonathan; University of the West of England, College of Health, Science and Society
**Primary Subject Heading**:	Emergency medicine
Secondary Subject Heading:	Emergency medicine, Health services research
Keywords:	ACCIDENT & EMERGENCY MEDICINE, TRAUMA MANAGEMENT, Systematic Review

SCHOLARONE™
Manuscripts

I, the Submitting Author has the right to grant and does grant on behalf of all authors of the Work (as defined in the below author licence), an exclusive licence and/or a non-exclusive licence for contributions from authors who are: i) UK Crown employees; ii) where BMJ has agreed a CC-BY licence shall apply, and/or iii) in accordance with the terms applicable for US Federal Government officers or employees acting as part of their official duties; on a worldwide, perpetual, irrevocable, royalty-free basis to BMJ Publishing Group Ltd ("BMJ") its licensees and where the relevant Journal is co-owned by BMJ to the co-owners of the Journal, to publish the Work in this journal and any other BMJ products and to exploit all rights, as set out in our licence.

The Submitting Author accepts and understands that any supply made under these terms is made by BMJ to the Submitting Author unless you are acting as an employee on behalf of your employer or a postgraduate student of an affiliated institution which is paying any applicable article publishing charge ("APC") for Open Access articles. Where the Submitting Author wishes to make the Work available on an Open Access basis (and intends to pay the relevant APC), the terms of reuse of such Open Access shall be governed by a Creative Commons licence – details of these licences and which Creative Commons licence will apply to this Work are set out in our licence referred to above.

Other than as permitted in any relevant BMJ Author's Self Archiving Policies, I confirm this Work has not been accepted for publication elsewhere, is not being considered for publication elsewhere and does not duplicate material already published. I confirm all authors consent to publication of this Work and authorise the granting of this licence.

For peer review only

Factors that influence the administration of tranexamic acid (TXA) to trauma patients in pre-hospital settings: A systematic review.

Helen Nicholson[1], Natalie Scotney[2], Simon Briscoe[3], Kim Kirby[1,2], Adam Bedson[2], Laura Goodwin[1], Maria Robinson[2], Hazel Taylor[4], Jo Thompson-Coon[5], Sarah Voss[1] & Jonathan Benger[1].

[1] College of Health, Science and Society, University of the West of England, Glenside Campus (1H14), Blackberry Hill, Bristol, BS16 1DD, England

[2] South Western Ambulance Service NHS Foundation Trust, Eagle Way, Exeter, EX2 7HY, England

[3] University of Exeter Medical School. University of Exeter, EX1 2LU

[4] Research Design Service – South West, University Hospitals Bristol NHS Foundation Trust, Upper Maudlin Street, Bristol, BS2 8AE, England

[5]NIHR Applied Research Collaboration South West Peninsula (PenARC), University of Exeter Medical School, University of Exeter, EX1 2LU

*corresponding author, email address: Helen5.Nicholson@uwe.ac.uk, Twitter: @EllisonHelen, ORCID ID: https://orcid.org/0000-0002-8964-0607

KEYWORDS: Tranexamic acid, emergency medical services, pre-hospital, hemorrhage, trauma management, systematic review.

ABSTRACT

Objective: In the United Kingdom (UK) there are around 5,400 deaths annually from injury. Tranexamic acid (TXA) prevents bleeding and has been shown to reduce trauma mortality. However, only 5% of UK major trauma patients who are at risk of haemorrhage receive pre-hospital TXA. This review aims to examine the evidence regarding factors influencing the pre-hospital administration of TXA to trauma patients.

Design: Systematic literature review.

Data Sources: AMED, CENTRAL, CINAHL, Cochrane Database of Systematic Reviews, Conference Proceedings Citation Index – Science, Embase and MEDLINE were searched from January 2010 to 2020; searches were updated in June 2022. Clinicaltrials.gov and OpenGrey were also searched and forwards and backwards citation chasing performed.

Eligibility Criteria: All primary research reporting factors influencing TXA administration to trauma patients in the pre-hospital setting was included.

Data Extraction and Synthesis: Two independent reviewers performed the selection process, quality assessment and data extraction. Data was tabulated, grouped by setting and influencing factor and synthesised narratively.

Results: Twenty papers (278,249 participants in total) were included in the final synthesis; 13 papers from civilian and 7 from military settings. Thirteen studies were rated as 'moderate' using the Effective Public Health Practice Project Quality Assessment Tool. Several common factors were

For peer review only

identified: knowledge and skills; consequences and social influences; injury type (severity, injury site and mechanism); protocols; resources; priorities; patient age; patient sex.

Conclusions: This review highlights an absence of high-quality research. Preliminary evidence suggests a host of system and individual-level factors that may be important in determining whether TXA is administered to trauma patients in the pre-hospital setting.

Funding and registration: This review was supported by Research Capability Funding from the South Western Ambulance Service NHS Foundation Trust and the National Institute for Health Research Applied Research Collaboration South West Peninsula.
PROSPERO registration number CRD42020162943 (protocol available online).

STRENGTHS AND LIMITATIONS OF THIS STUDY

- Strengths of this review include the extensive searches, the use of best practice methods and tools, reporting according to PRISMA guidelines and the input of a multi-disciplinary study team.
- Generalisability was enhanced by the separation of civilian and military studies.
- The review was limited by the small number of relevant studies, which precluded a formal synthesis as well as an English language restriction (two studies excluded due to language).
- In a deviation from the protocol, we were unable to search the ICTRP Search Portal. However, we were able to search the ClinicalTrials.gov registry which did not identify any new studies for inclusion; we therefore believe the impact of this was minimal.

INTRODUCTION

At least 20,000 individuals are severely injured in England each year, resulting in approximately 5,400 deaths, of which 2,400 occur before hospital arrival[1]. The CRASH2 trial provided strong evidence of the effectiveness of tranexamic acid (TXA) in reducing mortality in bleeding trauma patients[2], with CRASH3 then extending its use to isolated head injuries[3]. When given within three hours of injury, TXA reduces death from bleeding in all types of trauma[4]. An additional exploratory analysis concluded that the intervention is more effective if administered as soon as possible, particularly within one hour[5]. In their meta-analysis of the CRASH2 and WOMAN trials, Gayet-Ageron et al. found that immediate treatment improved survival by more than 70%, and that the survival benefit decreased by 10% for every 15 minutes of delay[6].

In 2011 South Western Ambulance Service NHS Foundation Trust was the first Emergency Medical Services (EMS) Provider (known as ambulance services in England) to implement the recommendations of CRASH2[7]; TXA was subsequently introduced across all UK ambulance services. However, analysis of UK Trauma Audit and Research Network (TARN) data found that only 5% of UK major trauma patients who are at risk of haemorrhage receive pre-hospital TXA[8]. The percentage of patients given TXA within one hour varies, with a reported range of 30% to 59%[9,10].

The timing of TXA administration is the single factor that determines effectiveness[4,5], and the key determinant of time to administration is the setting in which it is given[10]. TARN data show that when administered pre-hospital, median time to TXA administration is 49 minutes, compared to a median time of 111 minutes when given in hospital[10].

Determining the full extent of a patient's injuries, and the associated bleeding risk, in the early stages of pre-hospital care can be challenging. This systematic review aims to examine evidence

For peer review only

regarding factors that influence the timely administration of TXA to pre-hospital trauma patients. Due to the paucity of literature on this subject from UK civilian pre-hospital practice this review included both international and military-based pre-hospital care in order to maximise the identification of potential influencing factors. This approach was intended to give the greatest possible insights into TXA administration.

METHODS

Searches

We searched: AMED (via EBSCO); CENTRAL (via the Cochrane Library); CINAHL (via EBSCO); Cochrane Database of Systematic Reviews (via the Cochrane Library); Conference Proceedings Citation Index – Science (via Web of Science); Embase (via Ovid); and MEDLINE (via Ovid). The search strategy was developed using MEDLINE by an information specialist (SB) in consultation with the team. It included a sensitivity maximising search filter for identifying studies on EMS staff (paramedics)[11] and the final version was tested using pre-identified studies to ensure it retrieved all known relevant research. Searches were conducted on 10/01/2020 with a date limit of 2010 (reflecting the publication date of CRASH-2[2], and most recently updated on 08/06/2022. Search results were exported to Endnote X8 and de-duplicated. The search strategies for each bibliographic database are reported in Appendix 1.

We supplemented these searches using ClinicalTrials.gov (see Appendix 1 for search terms), checking reference lists, forward citation searching and OpenGrey (www.opengrey.eu). Forward citation searching used the Science Citation Index (via Web of Science) on 07/07/2020, 29/01/2021 and 13/12/2022. If an included study was not indexed in the Science Citation Index we used either Scopus or Google Scholar. Reference lists of included studies were checked manually.

Article selection

All primary pre-hospital research involving trauma patients and detailing any factors influencing TXA administration was included (see Appendix 2 for inclusion and exclusion criteria). Due to resource constraints the review was limited to English language publications. Study titles and abstracts were screened by two independent reviewers (HN and NK/KK). Disagreements were resolved through arbitration by a third reviewer (SV). The full text of all articles that appeared to meet eligibility criteria were retrieved. Full texts were assessed following the same process as the title and abstract screening. All eligible papers proceeded to data extraction.

Quality Appraisal

The Quality Assessment Tool for Quantitative Studies from the Effective Public Health Practice Project (EPHPP) was independently used by two reviewers (HN and NK/KK) to assess quantitative studies for risk of bias, study design, confounders and results[12,13]. Studies were graded 1-3 (strong, moderate or weak). Qualitative studies were assessed using the Critical Appraisal Skills Programme (CASP) Qualitative Research Checklist[14].

Studies were not excluded on the basis of poor quality, but methodological limitations and their effect on confidence in the findings of each paper were considered when drawing conclusions[15].

Data collection and synthesis

For peer review only

Data was extracted by two independent reviewers (HN and NK/KK) using a pre-determined data collection table (see Appendix 3 for data collection variables). Studies were tabulated and grouped by influencing factors and described with the aim of identifying patterns.

Patient and Public Involvement

No patient involved.

RESULTS

The PRISMA diagram is shown in Appendix 4. Twenty papers were included in the final synthesis. In order to aid interpretation, the papers were divided into civilian (13 papers) and military settings (7 papers) (Table 1 & Appendix 5 and Table 2 & Appendix 6 respectively). All but one of the papers were quantitative in design. Thirteen of the studies were retrospective observational or service evaluation studies, with four observational cohort studies[16–19], one case control study[20], one case series[21] and one qualitative interview study[22].

Thirteen studies were rated as 'moderate' using the EPHPP Quality Assessment Tool. One study was rated 'strong'[20] due to its case-control design. One abstract[23] was rated 'weak' due to a lack of published information and four other papers were rated 'weak' as a result of study design and data collection methods[17–19,24] . The qualitative study was rated as 'valuable' using the CASP Qualitative Research Checklist[14]. All but five of the studies were retrospective, and data collection was often incomplete. However, all of the studies explained patient dropouts or exclusions except the abstract[23].

Only two of the papers included paediatric patients in their studies[9,25] and three of the papers limited their studies to adult trauma patients with severe traumatic brain injury[17–19]. Only the Goodwin et al. paper had primary outcomes relating directly to factors influencing the pre-hospital administration of TXA[22]. This was a qualitative study interviewing 18 UK paramedics from one ambulance service about the barriers and facilitators of TXA administration. Using the factors reported from this paper and the reported outcomes from the other papers it was possible to identify several factors of interest: knowledge and skills; consequences and social influences; injury type (including severity, injury site and mechanism of injury); protocols; resources; priorities; patient age; patient sex.

For peer review only

Table 1. Summary table of characteristics of included civilian studies (n=13) (see Appendix 5 for full table).

First Author (date), country	Factors influencing TXA administration
Bossers (2021), Netherlands	**Patient age** - patients receiving TXA older than those not receiving TXA (47 vs 45 years).<br>**Injury type, severity -** patients receiving TXA had higher Injury Severity Score (ISS) (27 vs 26), lower prehospital Glasgow Coma Score (GCS) (4 vs 5) and higher heart rate (98 vs 92).
El-Menyar (2020), Qatar	**Resources** – patients did not receive TXA if critical care paramedics unavailable.
Goodwin (2021), UK	**Knowledge and skills** - inadequate training and a lack of knowledge of the effects of TXA or the evidence base behind its use and a lack of exposure to trauma patients were barriers to its administration.<br>**Resources -** a lack of time and staffing was a barrier to administration. Helicopter Emergency Medicine Services (HEMS) attendance was a barrier to some as they preferred to wait for a HEMS team member to administer it.<br>**Protocol -** guidelines felt restrictive or confusing. Disparity between paramedic, HEMS and doctor TXA protocols causes confusion. The drug preparation and administration route were seen as barriers to its use.<br>**Consequences and social influences-** the benefits of TXA were seen to outweigh the risks. TXA was seen to signal a major trauma patient. Fear of repercussion for administering TXA inappropriately. Opinion of colleagues seen to influence TXA administration.<br>**Priorities** - TXA not seen as a priority, with administering fluids or distracting injuries often taking precedent. Three-hour window of administration may reduce the perceived urgency of administration. The stress associated with trauma jobs may lead to TXA being overlooked or deprioritised.<br>**Injury type, severity -** risk of bleeding influenced administration including clinical observations and patient presentation. Participants found identifying patients at risk of bleeding difficult.<br>**Injury type, Mechanism of Injury (MOI) -** uncertainty over which injuries/conditions TXA is indicated for. MOI and type of injury were part of identify patients at risk of bleeding.<br>**Injury type, site** - less obvious bleeding including occult/internal haemorrhage was harder to identify.
Marsden (2019), UK	**Injury type, MOI** – road traffic collisions more likely to receive TXA, falls less likely.<br>**Injury type, severity** – patients given TXA more likely to have heart rate and blood pressure suggestive of bleeding.<br>**Resources** – patients given TXA were more likely to be treated by a physician-led crew.
McQueen (2013), UK	**Protocol** - clinician judgement used to guide administration outside of the protocol.<br>**Injury type, MOI** – most TXA patients had multiple injuries.<br>**Resources** - time constraints on scene and the absence of a doctor as part of the HEMS crew meant TXA not given.
Neeki (2018), USA	**Resources** - not all Emergency Medicine Service providers carrying TXA.
Ng (2018), Canada	**Injury type, severity** – more patients receiving TXA had moderate (9-12) or severe (3-8) GCS scores than those not receiving TXA.

For peer review only

Nutbeam (2022), UK	**Sex** - female patients less likely to receive TXA (OR 0.35, 95% CI, 0.33-0.36).<br>**Injury type, severity** - females less likely than males to receive TXA in all ISS categories.<br>**Injury type, MOI** - females less likely than males to receive TXA for all injury mechanisms except motor vehicle crashes.
van Wessem (2022), Netherlands	**Patient age** - patients receiving TXA younger than those not receiving TXA (42 vs 53 years).<br>**Injury type, severity -** patients needing pre-hospital intubation, urgent laparotomy or with more deranged physiology more likely to have received TXA.
van Wessem (2021), Netherlands	**Patient age** - patients receiving TXA younger than those not receiving TXA (41 vs 51 years).<br>**Injury type, severity -** patients receiving TXA slightly more severely injured, had higher Abbreviated Injury Scale (AIS) Head scores and more often prehospitally intubated.
Vu (2013), USA	**Priorities** - balancing critical interventions, resuscitation and short flight times meant some eligible patients did not receive TXA.
Wafaisade (2016), Germany	**Injury type, severity** - patients needing pre-hospital intubation or chest tube placement more likely to receive TXA.<br>**Injury type, site** - patients with AIS ≥3 for abdomen or extremities more likely to receive TXA. Patients with AIS ≥3 for head or thorax less likely to receive TXA.<br>**Age** - patients over 60 years old less likely to receive TXA.
Wong (2021), Canada	**Age** - TXA group notably younger (38.2 years vs 49.1 years)<br>**Injury type, severity** - TXA group had higher mean ISS and more patients with hypotension.<br>**Resources** - more patients receiving TXA had a paramedic of higher certification level in attendance than those who did not receive TXA.

Table 2. Summary table of characteristics of included military setting studies (n=7). (see Appendix 6 for full table).

First Author (date), country	Factors influencing TXA administration
Benov (2019), Israel-Syrian Border	**Protocol** – TXA for penetrating torso injury regardless of haemodynamic status. All patients receiving freeze-dried plasma with a known time of injury <3 hours received TXA.<br>**Injury type, MOI** – majority of TXA patients had penetrating injury.
Fisher (2019), Iraq and Afghanistan	**Injury type, severity** – patients with higher ISS, tourniquet application or serious injuries to the thorax, abdomen, extremities and skin more likely to receive TXA.<br>**Injury type, MOI** - explosive injury and gunshot wounds were more likely to receive TXA. More likely to have explosive injuries and less likely gunshot wounds in TXA patients who had a tourniquet applied.<br>**Patient age** - amputation patients receiving TXA were younger than those not receiving TXA (22 vs 25 years).
Lipsky (2014), Israel	**Protocol** – 30% of TXA administrations had no clear indication for TXA administration. Altered level of consciousness mistakenly categorised as a sign of haemodynamic instability.<br>**Priorities** – some non-administrations due to tactical limitations, resuscitation or to avoid delaying evacuation.

For peer review only

Mahalo (2021), Israel	**Injury type, severity** - TXA administration associated with analgesic treatment.
Nadler (2014), Israel	**Protocol** – more conservative protocol in the civilian service compared to the military service but higher proportion of patients given TXA outside of protocol (with clearance) by civilian service.
Nadler (2021), Israel	**Protocol -** New Clinical Practice Guidelines (indicating TXA at a heart rate of 130 instead of 110) introduced caused a significant decrease in the proportion of TXA administered. Only 22% of patients indicated for TXA received it.
Tsur (2020), Israel	**Injury type, site** – isolated neck injuries more likely to receive TXA than no-neck injuries.

Injury type

This factor was the most prevalent (14/19 papers)[9,17–19,22–24,26–32] and could be further divided into injury severity, injury site and mechanism of injury.

Injury severity: Patients with a higher Injury Severity Score (ISS) were more likely to receive TXA in both military and civilian settings[17,24,29]. This was also the case within various sub-cohorts of combat casualties, such as those who had a tourniquet applied or with gunshot wounds[29]. Civilian patients needing pre-hospital tracheal intubation[18,19] or chest tube placement were also more likely to receive TXA[32]. However, one paper found no difference in ISS between patients that received pre-hospital and in-hospital TXA[9]. Mahalo et al. found that TXA administration in combat casualties was associated with analgesic treatment[26].

Injury site: Injury location influenced TXA administration, with paramedics reporting that less obvious bleeding, such as internal haemorrhage, was harder to identify therefore making the administration of TXA less likely[22]. Combat casualties were more likely to receive TXA if they suffered an isolated neck injury (15%, compared to 4% without neck injuries: P=0.01)[31]. This may be due, in part, to the fact that the neck is mostly a non-compressible area, and therefore requires alternative haemorrhage control strategies. However, the number of isolated neck injury patients was small (41, compared to 3,185 without neck injuries over a 12-year period).

In one civilian study, when using the Abbreviated Injury Score (AIS) to assess trauma patients, a score of 3 or more for the head or thorax was associated with a significantly lower chance of receiving TXA, whereas for the abdomen or extremities it was associated with an increased chance[32]. However, van Wessem et al. found that patients receiving TXA had higher Abbreviated Injury Scale (AIS) Head scores[19].

Ng et al. found that more patients receiving TXA in a civilian setting had a moderately or severely reduced Glasgow Coma Scale (GCS) score compared to those not receiving TXA, although statistical significance was not reported[30]. This was supported by Bossers et al. who found that patients receiving TXA had a significantly lower pre-hospital GCS than those not receiving it[17].

Mechanism of injury: Patients with multiple injuries made up 72% of patients receiving TXA in one study[23]. Mechanism of injury influenced the likelihood of receiving pre-hospital TXA in some of the civilian studies. Marsden et al. compared those receiving pre-hospital TXA with those receiving it in hospital[9]. Mechanism of injury was a statistically significant factor, with pre-hospital clinicians more likely to recognise the need for TXA in road traffic collisions than in falls. Penetrating injury was a positive determinant of TXA administration even when protocols did not specifically prompt

For peer review only

this[9,20]. Penetrating trauma was also linked to TXA administration in combat casualties,[29,33] with patients sustaining explosive injury and gunshot wounds more likely to receive TXA[29]. Participants in the Goodwin et al. study confirmed that mechanism of injury played a part in identifying patients at risk of bleeding, however, they also reported uncertainty over which injuries or conditions TXA was indicated for[22].

Protocols

This factor encompassed both the contents of protocols (indications for use, route of administration) and the how they were used (compliance, confusion) and it was closely linked to injury type. Where described, the details of TXA protocols varied, but usually included exact indications (patient observation ranges and/or mechanism of injury)[18–21,24,25,33–35]. Recognised signs of clinical shock were most often the basis of these protocols[18–21,34]. In some, the presence of major trauma[21], the use of freeze-dried plasma[25], capillary refill time or altered level of consciousness were also included[34]. However, for the National Israeli Civilian Services only age (>18 years), expected evacuation times (>15 minutes) and the presence of a non-compressible haemorrhage were specified as indications[33]. The Tactical Combat Casualty Care guidelines used by the US military employ the more subjective indication of “anticipated to need significant blood transfusion”[29]. This was similar to the “clinical suspicion of major haemorrhage” or “clinical judgement” reported in the indications for TXA in some civilian studies[18,19,23].

This variation in protocol was also mentioned by participants in the Goodwin et al. study where they felt that the disparity between paramedic, helicopter emergency medical services (HEMS) and doctor TXA protocols caused confusion[22]. They reported that the guidelines for TXA use felt restrictive or confusing, and that the drug preparation and administration route were seen as barriers to its use.

The TXA protocol for the Israel Defence Force specifies use in patients receiving freeze-dried plasma as well as those with penetrating torso injury, regardless of haemodynamic status[25]. It is therefore unsurprising that 50.9% (n=113) who received TXA in the Benov et al. study presented with penetrating injury and tachycardia, and all patients known to be less than three hours from injury who received freeze-dried plasma also received TXA[25]. However, this study was limited by incomplete data collection and the assumption that severely injured patients would likely have died before reaching medical assistance.

Resources

Time, clinician and TXA availability appear to influence TXA administration[9,16,20,22,23]. In addition to twelve cases (48%) of TXA administrations being outside protocol, McQueen & Wyse noted a further thirteen (46%) indicated cases where TXA was not administered[23]. They identified time constraints on scene and the absence of a doctor as reasons for non-administration. El-Menyar et al. found that the time from EMS activation to hospital arrival was significantly longer in the TXA group (62 vs 74 minutes, p=0.03)[20]. This suggests that TXA administration is associated with increased pre-hospital time; either due to the requirements of drug administration, or additional procedures that patients who are given TXA require at scene.

The grade of clinician able to administer TXA varied. El-Menyar et al. reported that TXA could only be administered by critical care paramedics, and suggested that a lack of availability of these staff led to some eligible patients not receiving TXA[20]. Marsden et al. found that patients who were given TXA were more likely to be treated by a physician-led crew (60.5% vs 31.6%, p<0.001)[9] and Wong et al. found that patients receiving TXA were attended by a paramedic of higher certification level than

For peer review only

those who did not receive TXA[24]. However, some participants in the Goodwin et al. study reported that HEMS attendance was a barrier to TXA administration as they preferred to wait for a HEMS team member to administer it[22].

However, the most fundamental resource issue is the availability of the drug itself, and despite strong evidence of effectiveness some services did not carry TXA[16].

Priorities

The Goodwin et al. study found that TXA was not seen as a priority by the paramedics interviewed with fluid administration or the treatment of injuries often taking precedent[22]. Some studies also suggested that other considerations may take priority over TXA administration. For example, Vu et al. found several patients who met the criteria but did not receive TXA, although the exact number was not disclosed[21]. In these cases, clinicians reported other priorities that required their attention as they balanced critical interventions, resuscitation and short flight times[21]. A similar situation was described by Lipsky et al. who found that some non-administrations may have been due to tactical limitations, resuscitation or to avoid delaying evacuation[34]. However, they also suggested that raising awareness amongst staff may prove beneficial.

The participants in the Goodwin et al. study also suggested that the 3-hour window may reduce the perceived urgency of TXA administration and that the stress associated with trauma patients may lead to TXA being overlooked or deprioritised[22].

Patient age

There was some evidence that a patient's age influenced administration, with patients over 60 years less likely to receive TXA in one study[32] and patients receiving TXA being notably younger (38.2 years vs 49.1 years) than those who did not in another study[24]. The van Wessem studies also found that trauma patients with severe traumatic brain injury receiving TXA were younger than those who did not (42 vs 53 years and 41 vs 51 years), although the seven years of data from the 2021 study were reused but extended by 6 months in the 2022 study meaning these should not be considered separate results[18,19]. However, Bossers et al. found that patients with severe traumatic brain injury receiving TXA were slightly older than those not receiving it (47 vs 45 years)[17].

In military settings Fisher et al. found that, of those with amputations, patients receiving TXA were younger than those not receiving TXA (22 vs 25 years, $p<0.001$)[29], though some of these differences are of questionable clinical significance.

Patient sex

Nutbeam et al. was the only paper to report a patient sex difference in TXA administration[27]. They found that female patients in all risk categories and age groups were less likely to receive TXA (OR 0.35, 95% CI, 0.33-0.36). This difference in receipt of TXA increased with increasing age and decreased as injury severity increased. Females were found to be less likely to receive TXA for all mechanisms of injury except motor vehicle crashes.

Knowledge and Skills

The participants in the Goodwin et al. study reported that inadequate training and a lack of knowledge of the effects of TXA or the evidence base behind its use were barriers to its administration[22]. A lack of exposure to trauma patients was also reported as a barrier to its administration.

For peer review only

Consequences and social influences

Paramedics interviewed in the Goodwin et al. study felt that the benefits of TXA outweigh the risks[22]. However, administering TXA was seen to signal a major trauma patient, leading to concerns about the acceptance of the patient at a major trauma centre or judgement from colleagues if it was given to less severely injured patients. The opinion of colleagues was seen as an influencing factor, with 7/18 paramedics reporting that they check with a senior colleague or hospital staff before administering TXA. Paramedics reported a fear of repercussion for administering TXA inappropriately. They also felt that a lack of visible effects on patients inhibited TXA use.

DISCUSSION

This review found a paucity of high-quality research addressing the factors that influence pre-hospital TXA administration. However, despite only one of the included papers having primary outcomes relating directly to factors influencing the pre-hospital administration of TXA, it was possible to identify several factors from the studies that suggested a host of system and individual-level factors that may be important in determining whether TXA is administered to trauma patients in the pre-hospital setting. Injury type and protocols were the most prominent factors.

Despite the fact that this systematic review is the most complete to date of factors influencing pre-hospital TXA administration, there are some limitations. Only two of the studies[9,25] included paediatric patients, and the number of children in each were low. Therefore, it is not possible to identify factors influencing the administration of TXA to children in this review. There was also variability between the protocols and resources available to pre-hospital clinicians as well as settings in the included studies.

Seven of the papers reported on patients from combat settings[25,26,29,31,33–35]. Although much has been learnt about trauma care from military medicine, these studies are less likely to be generalisable to civilian trauma, and included disproportionately high numbers of young men and penetrating trauma. Despite this, the influencing factors identified were in line with those found in the civilian studies. However, in the military studies protocol and injury type seemed to play a more prominent role. Generalisability of the review was aided by the separation of civilian and military studies. Of the seven combat-specific studies, six were based on the experiences of the same pre-hospital service and included considerable author, data and date range overlap. A similar issue was found for the two studies from the Netherlands[18,19]. This raises the issue of potential pseudo-replication within the review. However, strengths of this review include the extensive searches, the use of PRISMA methods and tools and the input of a multi-disciplinary study team.

Protocols played an understandably important role in TXA administration[25]. However, criteria for administration and protocol compliance varied. In general protocol compliance was poor, suggesting that alternative factors play a part in clinical decision-making. This variability in protocol compliance results in both under administration and inappropriate administration of TXA[9,24,30,35–37]. Goodwin et al. found that confusion and restrictions relating to guidelines for TXA administration were barriers to administration[22]. Lipsky et al. reported that 30% of TXA administrations were for patients with no clear indication[34]. They found that in some of these cases altered levels of consciousness from blunt head injury appeared to be mistaken for haemodynamic instability. McQueen & Wyse reported that 48% of TXA administrations did not fulfil the inclusion criteria in their protocol[23], which they attributed to the influence of clinician judgement. However, a more subjective guideline relying on a clinician’s ability to identify patients likely to require significant

For peer review only - http://bmjopen.bmj.com/site/about/guidelines.xhtml

blood transfusion resulted in only 4.2% of those given TXA receiving ≥10 units of blood within the following 24 hours[29]. This suggests that clinical judgement may not be the most effective approach to guiding TXA administration, given that it can result in both low administration rates and high levels of inappropriate use.

It therefore seems preferable to focus on more objective protocols combined with improved compliance. Nadler et al. found that only 22% of patients indicated for TXA in a military setting received it[35]. In a civilian setting Wong et al. found that 35% of eligible patients had received pre-hospital TXA and reported that this was higher than other rates reported in Canada[24]. They suggested that factors such as poor intra-venous accessibility, time constraints or short transport times might limit willingness to administer TXA. Naddler et al. suggested that significant over and under treatment of TXA may indicate insufficient professionalism or insufficient knowledge of the practice guidelines[35]. Other papers suggested ways to improve compliance including raising awareness, improved training and the use of a decision-making tool to aid in identifying a patient's bleeding risk[22,34]. However, the inclusion of training on TXA administration is not always mandated, even within combat medical training, and compliance with training can be low[29].

The most frequent factors mentioned in protocols were signs of clinical shock, penetrating injury and administration of other treatments. It is not surprising that patients who receive pre-hospital TXA tend to have more overt haemodynamic instability[9]. This may be a product of pre-hospital protocols that tend to be weighted towards signs of clinical shock as a key criterion for TXA administration.

The lack of obvious benefits at the time of administration was suggested as a relevant factor in the study by Goodwin et al.[22]. Interestingly, Lipsky et al. included the fact that survival benefit is only seen after 24-48 hours in their protocol training materials[34]. They suggest informing clinicians that they should not expect improved field haemorrhage control following TXA. It is possible that making this clear in training materials or protocols may help to address one potential barrier to TXA administration. Another factor that may contribute to low administration rates is the need for slow intravenous infusion of TXA. Clinicians in the Fisher et al. study were required to administer TXA using a 10-minute infusion[29]. The authors suggest this may not be compatible with a tactical situation and propose that oral or intramuscular TXA may help to increase administration rates. The requirement for slow intravenous administration was also found to be a barrier in the study by Goodwin et al.[22]. Participants noted that the preparation of the drug (drawn up from two vials for an adult dose) could be a barrier in a time pressured situation.

Age was also found to influence TXA administration with younger patients generally more likely to receive TXA[18,19,24,29,32], although the difference in age was often of questionable clinical significance. However, the evidence around a sex difference in administration was very clear[27]. Female patients are less likely to be treated with TXA in all risk categories despite TXA reducing trauma deaths to a similar extent in men and women. The disparity was greatest in older women and women at lower risk.

Following the 2019 CRASH3 study and subsequent sub analyses[3], TXA has been indicated in the UK for adult patients with isolated traumatic head injury (CGS score of 12 or less) since July 2020[38]. However, all of the studies in this review used data predominantly predating the reporting of CRASH3. The Nutbeam et al. data did briefly overlap the CRASH3 publication, but it did not specifically report on head injuries[27]. The two van Wessem papers also briefly overlapped but they specifically excluded patients with isolated head injuries[18,19]. Of the three papers looking at trauma patients with severe brain injury all three showed patient age and the severity of injury to be

For peer review only

influencing factors, with more severely injured patients more likely to receive TXA[17–19]. However, the influence of age on this patient group was mixed and requires further study. It seems there is a lack of evidence around the factors influencing TXA administration in patients with severe head injuries and in the case of isolated head injury this may be due to the relatively recent changes to protocols around TXA administration for these patients.

Other important barriers to administration identified by Goodwin et al. included a lack of knowledge and experience with TXA, a lack of resources and difficulty in identifying patients at risk of bleeding[22]. These findings may help to explain the low protocol compliance identified in this review, although none of the studies examined causal factors in detail. Injury severity played a role in administration, and although patients who are more severely injured are more likely to receive TXA[17,24,29,39], the measure used to assess injury severity in the included studies was often the ISS, which can only be calculated accurately following hospital assessment. Therefore, it is unclear what measure of severity pre-hospital clinicians are using to determine the need for TXA administration, and this may introduce additional variability.

A new scoring system to identify trauma patients at risk of bleeding has recently been published. This showed that the proportion of patients receiving pre-hospital TXA could be increased through the use of the Bleeding Audit Triage Trauma (BATT) score[8]. The BATT score is calculated using a weighted system including age, systolic blood pressure, GCS, respiratory rate, heart rate and whether the injury was penetrating or involved high velocity trauma. This adds weight to the idea that clinical observations are commonly used, and potentially effective, in guiding more accurate pre-hospital TXA administration. A challenge that emerges is how to help pre-hospital clinicians identify and treat not just patients at highest risk of bleeding following trauma, but also those at lower risk who may nevertheless benefit from TXA administration, given that the drug can be safely administered to a wide spectrum of patients with traumatic bleeding, and should not be restricted to the most severely injured[40]. The newly validated pre-hospital BATT score shows promise in helping to identify these lower risk patients[8]. Treating patients with a BATT score of just 2 or more (out of 27) would result in 26 fewer deaths per 10,000 trauma patients.

CONCLUSIONS

This review highlights a lack of high-quality research addressing the factors that influence pre-hospital TXA administration, particularly in children or patients with isolated head injuries. Common factors identified in this review suggest a host of system and individual-level factors that may be important in determining whether TXA is administered to trauma patients in the pre-hospital setting. These include: knowledge and skills; consequences and social influences; injury type (including severity, injury site and mechanism of injury); protocols; resources; priorities; patient age; patient sex. Despite the well-established benefits of TXA in trauma, and the central role of time to administration[4,5], pre-hospital administration rates remain low[9,29]. In order to address this evidence-practice gap it is essential that the factors influencing pre-hospital administration are better understood.

ABBREVIATIONS

AIS: Abbreviated Injury Scale

BATT: Bleeding Audit Triage Trauma

EMS: Emergency Medical Services

For peer review only

GCS: Glasgow Coma Score

HEMS: Helicopter Emergency Medical Services

ICTRP: International Clinical Trials Registry Platform

IDF: Israeli Defence Force

ISS: Injury Severity Score

EPHPP: Effective Public Health Practice Project

TARN: Trauma Audit and Research Network

TXA: Tranexamic Acid

UK: United Kingdom

DECLARATIONS

Ethics approval and consent to participate

Not applicable

Consent for publication

Not applicable

Availability of data and materials

The datasets generated and analysed during the review are not publicly available but are available from the corresponding author on reasonable request.

Competing interests

The authors declare that they have no competing interests.

Funding

This review was supported by Research Capability Funding from the South Western Ambulance Service NHS Foundation Trust and the National Institute for Health Research Applied Research Collaboration South West Peninsula (CE-18-1497/HAS-NAM-18-026). The views expressed in this publication are those of the authors and not necessarily those of the National Institute for Health Research of the Department of Health and Social Care.

Author contributions

HN participated in review conception, design and coordination, searches, paper selection, quality assessment, data extraction, data synthesis and drafted the manuscript. NS participated in searches, paper selection, quality assessment and data extraction. KK participated in conception, design, paper selection, quality assessment and data extraction. SV participated in conception, design and coordination and paper selection. JB, MR, AB, LG and HT participated in conception and design. JTC participated in design and data synthesis. SB participated in design and undertook database searches. All authors were responsible for critical revision of the manuscript for publication and approved the final version to be published.

Acknowledgements

For peer review only

We would like to thank Professor Ian Roberts (London School of Hygiene & Tropical Medicine), Professor Tim Coats (University of Leicester) and Dr Francois-Xavier Ageron (University of Lausanne) for their assistance during this project.

Registration
PROSPERO registration number CRD42020162943.

REFERENCES

1 National Audit Office. Major Trauma care in England. 2010.

2 Olldashi F, Kerçi M, Zhurda T, *et al.* Effects of tranexamic acid on death, vascular occlusive events, and blood transfusion in trauma patients with significant haemorrhage (CRASH-2): A randomised, placebo-controlled trial. *The Lancet* 2010;**376**:23–32. doi:10.1016/S0140-6736(10)60835-5

3 Roberts I, Shakur-Still H, Aeron-Thomas A, *et al.* Effects of tranexamic acid on death, disability, vascular occlusive events and other morbidities in patients with acute traumatic brain injury (CRASH-3): A randomised, placebo-controlled trial. *The Lancet* 2019;**394**:1713–23. doi:10.1016/S0140-6736(19)32233-0

4 Roberts I, Edwards P, Prieto D, *et al.* Tranexamic acid in bleeding trauma patients: An exploration of benefits and harms. *Trials* 2017;**18**:48. doi:10.1186/s13063-016-1750-1

5 Olldashi F, Kerçi M, Zhurda T, *et al.* The importance of early treatment with tranexamic acid in bleeding trauma patients: An exploratory analysis of the CRASH-2 randomised controlled trial. *The Lancet* 2011;**377**:1101.e1-1101.e2. doi:10.1016/S0140-6736(11)60278-X

6 Gayet-Ageron A, Prieto-Merino D, Ker K, *et al.* Effect of treatment delay on the effectiveness and safety of antifibrinolytics in acute severe haemorrhage: a meta-analysis of individual patient-level data from 40 138 bleeding patients. *The Lancet* 2018;**391**:125–32. doi:10.1016/S0140-6736(17)32455-8

7 Paudyal P, Smith J, Robinson M, *et al.* Tranexamic acid in major trauma: implementation and evaluation across South West England. *Eur J Emerg Med* 2017;**24**:44–8.

8 Ageron FX, Coats TJ, Darioli V, *et al.* Validation of the BATT score for prehospital risk stratification of traumatic haemorrhagic death: usefulness for tranexamic acid treatment criteria. *Scand J Trauma Resusc Emerg Med* 2021;**29**:6. doi:10.1186/s13049-020-00827-5

9 Marsden MER, Rossetto A, Duffield CAB, *et al.* Prehospital tranexamic acid shortens the interval to administration by half in Major Trauma Networks: a service evaluation. *Emergency Medicine Journal* 2019;**36**:395–400. doi:10.1136/emermed-2018-208118

10 Coats TJ, Fragoso-Iñiguez M, Roberts I. Implementation of tranexamic acid for bleeding trauma patients: a longitudinal and cross-sectional study. *Emergency Medicine Journal* 2019;**36**:78–81. doi:10.1136/emermed-2018-207693

11 Olaussen A, Semple W, Oteir A, *et al.* Paramedic literature search filters: Optimised for clinicians and academics. *BMC Med Inform Decis Mak* 2017;**17**:146. doi:10.1186/s12911-017-0544-z

For peer review only

12 Quality Assessment Tool for Quantitative Studies. https://www.ephpp.ca/quality-assessment-tool-for-quantitative-studies/ (accessed 27 Jan 2021).

13 Armijo-Olivo S, Stiles CR, Hagen NA, *et al.* Assessment of study quality for systematic reviews: A comparison of the Cochrane Collaboration Risk of Bias Tool and the Effective Public Health Practice Project Quality Assessment Tool: Methodological research. *J Eval Clin Pract* 2012;**18**:12–8. doi:10.1111/j.1365-2753.2010.01516.x

14 CASP CHECKLISTS - CASP - Critical Appraisal Skills Programme. https://casp-uk.net/casp-tools-checklists/ (accessed 27 Jan 2021).

15 Greenfield K, Holley S, Schoth DE, *et al.* Barriers and facilitators experienced by patients, carers and healthcare professionals when managing symptoms in infants, children and young people at end-of-life: a mixed methods systematic review protocol. doi:10.1136/bmjopen-2019-030566

16 Neeki MM, Dong F, Toy J, *et al.* Tranexamic acid in civilian trauma care in the California Prehospital Antifibrinolytic Therapy study. *Western Journal of Emergency Medicine* 2018;**19**:977–86. doi:10.5811/westjem.2018.8.39336

17 Bossers SM, Loer SA, Bloemers FW, *et al.* Association between Prehospital Tranexamic Acid Administration and Outcomes of Severe Traumatic Brain Injury. *JAMA Neurol* 2021;**78**:328–45. doi:10.1001/jamaneurol.2020.4596

18 van Wessem KJP, Jochems D, Leenen LPH. The effect of prehospital tranexamic acid on outcome in polytrauma patients with associated severe brain injury. *European Journal of Trauma and Emergency Surgery* 2022;**48**:1589–99. doi:10.1007/s00068-021-01827-5

19 van Wessem KJP, Luke •, Leenen PH. Does Liberal Prehospital and In-Hospital Tranexamic Acid Influence Outcome in Severely Injured Patients? A Prospective Cohort Study. *World J Surg* 2021;**45**:2398–407. doi:10.1007/s00268

20 El-Menyar A, Sathian B, Wahlen BM, *et al.* Prehospital administration of tranexamic acid in trauma patients: A 1:1 matched comparative study from a level 1 trauma center. *American Journal of Emergency Medicine* 2020;**38**:266–71. doi:10.1016/j.ajem.2019.04.051

21 Vu EN, Schlamp RS, Wand RT, *et al.* Prehospital Use of Tranexamic Acid for Hemorrhagic Shock in Primary and Secondary Air Medical Evacuation. *Air Med J* 2013;**32**:289–92. doi:10.1016/j.amj.2013.05.001

22 Goodwin L, Nicholson H, Robinson M, *et al.* Barriers and facilitators to the administration of prehospital tranexamic acid: a paramedic interview study using the theoretical domains framework. *Emerg Med J* 2022;**39**:540–6. doi:10.1136/emermed-2020-210622

23 McQueen C, Wyse M. The introduction of Tranexamic Acid to Helicopter Emergency Medical Service operations in a regional service in the United Kingdom. *Trauma (United Kingdom)* 2013;**15 (4)**:344.https://ovidsp.ovid.com/ovidweb.cgi?T=JS&CSC=Y&NEWS=N&PAGE=fulltext&D=emed14&AN=71418011

24 Wong D, Su G, Mabasa VH, *et al.* Assessing the clinical utilization of tranexamic acid by paramedics for patients with major trauma (ACUTE). *Canadian Journal of Emergency Medicine* 2021;**23**:219–22. doi:10.1007/s43678-020-00040-4

For peer review only

25 Benov A, Shkolnik I, Glassberg E, *et al.* Prehospital Trauma Experience of the Israel Defense Forces on the Syrian Border 2013-2017. *J Trauma Acute Care Surg* Published Online First: 2019. doi:10.1097/TA.0000000000002217

26 Betelman Mahalo Y, Avital G, Radomislensky I, *et al.* Pain management for casualties receiving lifesaving interventions in the prehospital scenario: Raising awareness of our human nature. *J Trauma Acute Care Surg* 2021;**91**:S201–5. doi:10.1097/TA.0000000000003295

27 Nutbeam T, Roberts I, Weekes L, *et al.* Use of tranexamic acid in major trauma: a sex-disaggregated analysis of the Clinical Randomisation of an Antifibrinolytic in Significant Haemorrhage (CRASH-2 and CRASH-3) trials and UK trauma registry (Trauma and Audit Research Network) data. *Br J Anaesth* 2022;**129**:191–9. doi:10.1016/j.bja.2022.03.032

28 Benov A, Salas MM, Nakar H, *et al.* Battlefield pain management: A view of 17 years in Israel Defense Forces. *Journal of Trauma and Acute Care Surgery* 2017;**83**:S150–5. doi:10.1097/ta.0000000000001481

29 Fisher AD, Carius BM, April MD, *et al.* An Analysis of Adherence to Tactical Combat Casualty Care Guidelines for the Administration of Tranexamic Acid. *Journal of Emergency Medicine* 2019;**57**:646–52. doi:10.1016/j.jemermed.2019.08.027

30 Ng M, Perrott J, Burgess S. Evaluation of tranexamic acid in trauma patients: A retrospective quantitative analysis. *American Journal of Emergency Medicine* 2019;**37**:444–9. doi:10.1016/j.ajem.2018.06.010

31 Tsur N, Benov A, Nadler R, *et al.* Neck injuries - israel defense forces 20 years' experience. *Injury* Published Online First: 2020. doi:https://dx.doi.org/10.1016/j.injury.2020.09.042

32 Wafaisade A, Lefering R, Bouillon B, *et al.* Prehospital administration of tranexamic acid in trauma patients. *Crit Care* 2016;**20**:1–9. doi:10.1186/s13054-016-1322-5

33 Nadler R, Gendler S, Benov A, *et al.* Tranexamic acid at the point of injury: The Israeli combined civilian and military experience. *Journal of Trauma and Acute Care Surgery* 2014;**77**:S146–50. doi:10.1097/ta.0000000000000325

34 Lipsky AM, Abramovich A, Nadler R, *et al.* Tranexamic acid in the prehospital setting: Israel Defense Forces' initial experience. *Injury* 2014;**45**:66–70. doi:10.1016/j.injury.2013.08.025

35 Nadler R, Tsur AM, Lipsky AM, *et al.* Trends in combat casualty care following the publication of clinical practice guidelines. *J Trauma Acute Care Surg* 2021;**91**:S194–200. doi:10.1097/TA.0000000000003280

36 Schauer SG, April MD, Naylor JF, *et al.* QuikClot® Combat Gauze® Use by Ground Forces in Afghanistan The Prehospital Trauma Registry Experience. *J Spec Oper Med* 2016;**17**:101–6.

37 Godfrey BW, Martin A, Chestovich PJ, *et al.* Patients with multiple traumatic amputations: An analysis of operation enduring freedom joint theatre trauma registry data. *Injury* 2017;**48**:75–9. doi:10.1016/j.injury.2016.08.008

38 Head Injury Algorithm - aace.org.uk. https://aace.org.uk/jrcalc-updates-2019/head-injury-algorithm/ (accessed 24 Jan 2023).

For peer review only

39 Morrison JJ, Dubose JJ, Rasmussen TE, *et al.* Military application of tranexamic acid in trauma emergency resuscitation (MATTERs) study. *Archives of Surgery* 2012;**147**:113–9. doi:10.1001/archsurg.2011.287

40 Roberts I, Perel P, Prieto-Merino D, *et al.* Effect of tranexamic acid on mortality in patients with traumatic bleeding: Prespecified analysis of data from randomised controlled trial. *BMJ (Online)* 2012;**345**. doi:10.1136/bmj.e5839

For peer review only

APPENDIX 1- Literature search report

Bibliographic database searches

Database: AMED

Host: EBSCO

Data Parameters: n/a

Date Searched: 10/1/2020

Searcher: SB

Hits: 2

Strategy:

1. TI "tranexamic acid" OR AB "tranexamic acid"
2. TI TXA OR AB TXA
3. TI cyklokapron OR AB cyklokapron
4. 1 or 2 or 3

Notes: Date limited 2010 – to date

Database: CENTRAL

Host: Cochrane Library

Data Parameters: Issue 1 of 12, January 2020

Date Searched: 10/10/2020

Searcher: SB

Hits: 196

Strategy:

#1 ("tranexamic acid"):ti OR ("tranexamic acid"):ab

#2 (TXA):ti OR (TXA):ab

#3 (cyklokapron):ti OR (cyklokapron):ab

#4 MeSH descriptor: [Tranexamic Acid] this term only

#5 #1 or #2 or #3 or #4

#6 (trauma*):ti OR (trauma*):ab

#7 (paramedic* or ems or emt or prehospital or "pre hospital" or "first responder*" or "emergency medical technician*" or "emergency services" or Ambulance* or HEMS or "field triage" or "out-of-hospital"):ti OR (paramedic* or ems or emt or prehospital or "pre hospital" or "first

For peer review only

responder*" or "emergency medical technician*" or "emergency services" or Ambulance* or HEMS or "field triage" or "out-of-hospital"):ab

#8 MeSH descriptor: [Ambulances] this term only

#9 MeSH descriptor: [Emergency Medical Technicians] this term only

#10 MeSH descriptor: [Air Ambulances] this term only

#11 MeSH descriptor: [Emergency Medical Services] this term only

#12 #6 or #7 or #8 or #9 or #10 or #11

#13 #5 and #12

Notes: Date limited 2010 – 2020

Database: CINAHL
Host: EBSCO
Data Parameters: n/a
Date Searched: 13/1/2020
Searcher: SB
Hits: 313
Strategy:

1. TI "tranexamic acid" OR AB "tranexamic acid"
2. TI TXA OR AB TXA
3. TI cyklokapron OR AB cyklokapron
4. (MH "Tranexamic Acid")
5. TI trauma* OR AB trauma*
6. TI ((paramedic* or ems or emt or prehospital or "pre hospital" or "first responder*" or "emergency medical technician*" or "emergency services" or Ambulance* or HEMS or "field triage" or "out-of-hospital")) OR AB ((paramedic* or ems or emt or prehospital or "pre hospital" or "first responder*" or "emergency medical technician*" or "emergency services" or Ambulance* or HEMS or "field triage" or "out-of-hospital"))
7. (MH "Ambulances")
8. (MH "Aeromedical Transport")
9. (MH "Emergency Medical Services+")
10. S1 OR S2 OR S3 OR S4
11. S5 OR S6 OR S7 OR S8 OR S9
12. S10 AND S11

For peer review only

Notes: Date limited 2010 – to date; English language results only

Database: Cochrane Database of Systematic Reviews (CDSR)

Host: Cochrane Library

Data Parameters: Issue 1 of 12, January 2020

Date Searched: 10/10/2010

Searcher: SB

Hits: 6

Strategy: see CENTRAL search

Notes: Date limited 2010 – 2020

Database: Conference Proceedings Citation Index – Science (CPCI-S)

Host: Clarivate Analytics

Data Parameters: n/a

Date Searched: 10/1/2020

Searcher: SB

Hits: 86

Strategy:

1. TOPIC: ("tranexamic acid")
2. TOPIC: (TXA)
3. TOPIC: (cyklokapron)
4. #1 OR #2 OR #3
5. TOPIC: (trauma*)
6. TOPIC: (paramedic* or ems or emt or prehospital or "pre hospital" or "first responder*" or "emergency medical technician*" or "emergency services" or Ambulance* or HEMS or "field triage" or "out-of-hospital")
7. #5 OR #6
8. (#7 AND #4) AND LANGUAGE: (English) Indexes=CPCI-S Timespan=2010-2020

Database: Embase

Host: Ovid

Data Parameters: 1974 to 2020 January 09

Date Searched: 10/1/2020

Searcher: SB

Hits: 1250

Strategy:

For peer review only

1. "tranexamic acid".tw.
2. TXA.tw.
3. cyklokapron.tw.
4. tranexamic acid/
5. or/1-4
6. trauma*.tw.
7. (paramedic* or ems or emt or prehospital or "pre hospital" or "first responder*" or "emergency medical technician*" or "emergency services" or Ambulance* or HEMS or "field triage" or "out-of-hospital").tw.
8. exp ambulance/
9. rescue personnel/
10. air medical transport/
11. exp emergency health service/
12. or/6-11
13. 5 and 12
14. limit 13 to (english language and yr="2010 –Current")

Database: MEDLINE ALL

Host: Ovid

Data Parameters: 1946 to January 09, 2020

Date Searched: 10/1/2020

Searcher: SB

Hits: 513

Strategy:

1. "tranexamic acid".tw.
2. TXA.tw.
3. cyklokapron.tw.
4. Tranexamic Acid/
5. or/1-4
6. trauma*.tw.
7. (paramedic* or ems or emt or prehospital or "pre hospital" or "first responder*" or "emergency medical technician*" or "emergency services" or Ambulance* or HEMS or "field triage" or "out-of-hospital").tw.
8. Ambulances/
9. Emergency Medical Technicians/
10. Air Ambulances/

For peer review only

11. Emergency Medical Services/
12. or/6-11
13. 5 and 12
14. Limit 13 to (english language and yr="2010 –Current")

Table A1. Total number of hits per database and unique records

Database	Hits
AMED	2
CENTRAL	196
CINAHL	313
CDSR	6
CPCI-S	86
Embase	1250
MEDLINE ALL	513
**Total records**	**2366**
**Duplicate records**	**605**
**Unique records**	**1761**

OpenGrey search

URL: http://www.opengrey.eu/

Date Searched: 09/03/2021

Searcher: HN

Hits: 11

Strategy: "tranexamic acid" OR TXA

Clinical trials registry search

Registry: ClinicalTrials.gov

URL: https://clinicaltrials.gov/

Date Searched: 24/06/2020

Searcher: HN

Hits: 27

Strategy:

Intervention/treatment: "tranexamic acid" OR TXA OR cyklokapron

Title/Acronym: trauma OR prehospital OR "pre-hospital" OR emergency

For peer review only

Update searches

Bibliographic database searches

Database: AMED

Host: EBSCO

Data Parameters: n/a

Date Searched: 14/12/2020

Searcher: SB

Hits: 0

Strategy: see above (date limited 2020 to date of search)

Database: CENTRAL

Host: Cochrane Library

Data Parameters: Issue 12 of 12, December 2020

Date Searched: 14/12/2020

Searcher: SB

Hits: 65

Strategy: see above (date limited 2020 to date of search)

Database: CINAHL

Host: EBSCO

Data Parameters: n/a

Date Searched: 14/12/2020

Searcher: SB

Hits: 68

Strategy: see above (date limited 2020 to date of search)

Database: Cochrane Database of Systematic Reviews (CDSR)

Host: Cochrane Library

Data Parameters: Issue 12 of 12, December 2020

Date Searched: 14/12/2020

Searcher: SB

Hits: 0

Strategy: see above (date limited 2020 to date of search)

For peer review only

Database: Conference Proceedings Citation Index – Science (CPCI-S)

Host: Clarivate Analytics

Data Parameters: n/a

Date Searched: 14/12/2020

Searcher: SB

Hits: 7

Strategy: see above (date limited 2020 to date of search)

Database: Embase

Host: Ovid

Data Parameters: 1974 to 2020 December 11

Date Searched: 14/12/2020

Searcher: SB

Hits: 227

Strategy: see above (date limited 2020 to date of search)

Database: MEDLINE ALL

Host: Ovid

Data Parameters: 1946 to December 11, 2020

Date Searched: 14/12/2020

Searcher: SB

Hits: 132

Strategy: see above (date limited 2020 to date of search)

Table A2. Total number of hits per database and unique records

**Database**	**Hits**
AMED	0
CENTRAL	65
CINAHL	68
CDSR	0
CPCI-S	7
Embase	227
MEDLINE ALL	132
**Total records**	**499**
**Duplicate records**	**189**
**Unique records**	**310**

For peer review only

Clinical trials registry search

Registry: ClinicalTrials.gov

URL: https://clinicaltrials.gov/

Date Searched: 25/01/2021

Searcher: HN

Hits: 30

Strategy: see above

[Please note that the hits reported from each of the clinical trials registry search updates included duplicates from the previous searches. However, we also found that there were both new records as well as records from previous searches that were now missing or had since been removed from the registry. Therefore, the number reported in the PRISMA diagram (34) was the total number of unique records found over the three searches.]

Update searches 2

Bibliographic database searches

Database: AMED

Host: EBSCO

Data Parameters: n/a

Date Searched: 8/6/2022

Searcher: SB

Hits: 4

Strategy: see above (date limited 2020 to date of search)

Database: CENTRAL

Host: Cochrane Library

Data Parameters: Issue 5 of 12, May 2022

Date Searched: 8/6/2022

Searcher: SB

Hits: 132

Strategy: see above (date limited 2020 to date of search)

Database: CINAHL

Host: EBSCO

Data Parameters: n/a

Date Searched: 9/6/2022

Searcher: SB

For peer review only

Hits: 712

Strategy: see above (date limited 2020 to date of search)

Database: Cochrane Database of Systematic Reviews (CDSR)

Host: Cochrane Library

Data Parameters: Issue 6 of 12, June 2022

Date Searched: 8/6/2022

Searcher: SB

Hits: 0

Strategy: see above (date limited 2020 to date of search)

Database: Conference Proceedings Citation Index – Science (CPCI-S)

Host: Clarivate Analytics

Data Parameters: n/a

Date Searched: 8/6/2022

Searcher: SB

Hits: 15

Strategy: see above (date limited 2020 to date of search)

Database: Embase

Host: Ovid

Data Parameters: 1974 to 2022 June 07

Date Searched: 8/6/2022

Searcher: SB

Hits: 571

Strategy: see above (date limited 2020 to date of search)

Database: MEDLINE ALL

Host: Ovid

Data Parameters: 1946 to June 07, 2022

Date Searched: 8/6/2022

Searcher: SB

Hits: 306

Strategy: see above (date limited 2020 to date of search)

Table A3. Total number of hits per database and unique records

For peer review only

Database	Hits
AMED	4
CENTRAL	132
CINAHL	712
CDSR	0
CPCI-S	15
Embase	571
MEDLINE ALL	306
**Total records**	**1740**
**Duplicate records**	**473**
**Duplicate record with previous search**	**222**
**Unique records**	**1045**

OpenGrey search

URL: http://www.opengrey.eu/

Date Searched: 25/01/2021

Searcher: HN

Hits: 18

Strategy: "tranexamic acid" OR TXA

Clinical trials registry search

Registry: ClinicalTrials.gov

URL: https://clinicaltrials.gov/

Date Searched: 25/01/2021

Searcher: HN

Hits: 33

Strategy: see above

[Please note that the hits reported from each of the clinical trials registry search updates included duplicates from the previous searches. However, we also found that there were both new records as well as records from previous searches that were now missing or had since been removed from the registry. Therefore, the number reported in the PRISMA diagram (34) was the total number of unique records found over the three searches.]

For peer review only

APPENDIX 2 - Inclusion and exclusion criteria

**PEOS categories**	**Inclusion criteria**	**Exclusion criteria**
**Population**	Any patients who have experienced traumatic injury	Patients receiving TXA who have not experienced traumatic injury or who have superficial minor wounds.<br><br>Patients experiencing obstetric, post-partum haemorrhage.
**Exposure**	Factors influencing the decision to administer TXA.	
**Outcome**	Papers detailing the characteristics of patients who did or did not receive TXA in the pre-hospital setting.<br><br>Papers detailing characteristics of pre-hospital clinicians who administered TXA.	TXA administration in hospital. Studies that solely report the clinical and/or cost effectiveness of TXA, dose or route of administration rather than the factors that influenced administration will be excluded.
**Setting**	Any pre-hospital setting where patients are treated by pre-hospital clinicians outside of a hospital or primary care environment.	Any hospital or primary care setting.
**Study design**	Any primary research.	Review articles, editorials and letters.

For peer review only

APPENDIX 3 – Variable sought during data extraction

List of variables for which data was sought during data extraction:

- Citation
- Country
- Study aims
- Patient and clinician population characteristics
- Primary outcome
- Inclusion and exclusion criteria
- Recruitment method
- Study Design
- Sample size and sampling method
- Data source
- Intervention and comparator group (if applicable)
- Date and duration of data collection
- Setting
- Analysis
- Factors influencing TXA administration

For peer review only

APPENDIX 4 – PRISMA diagram

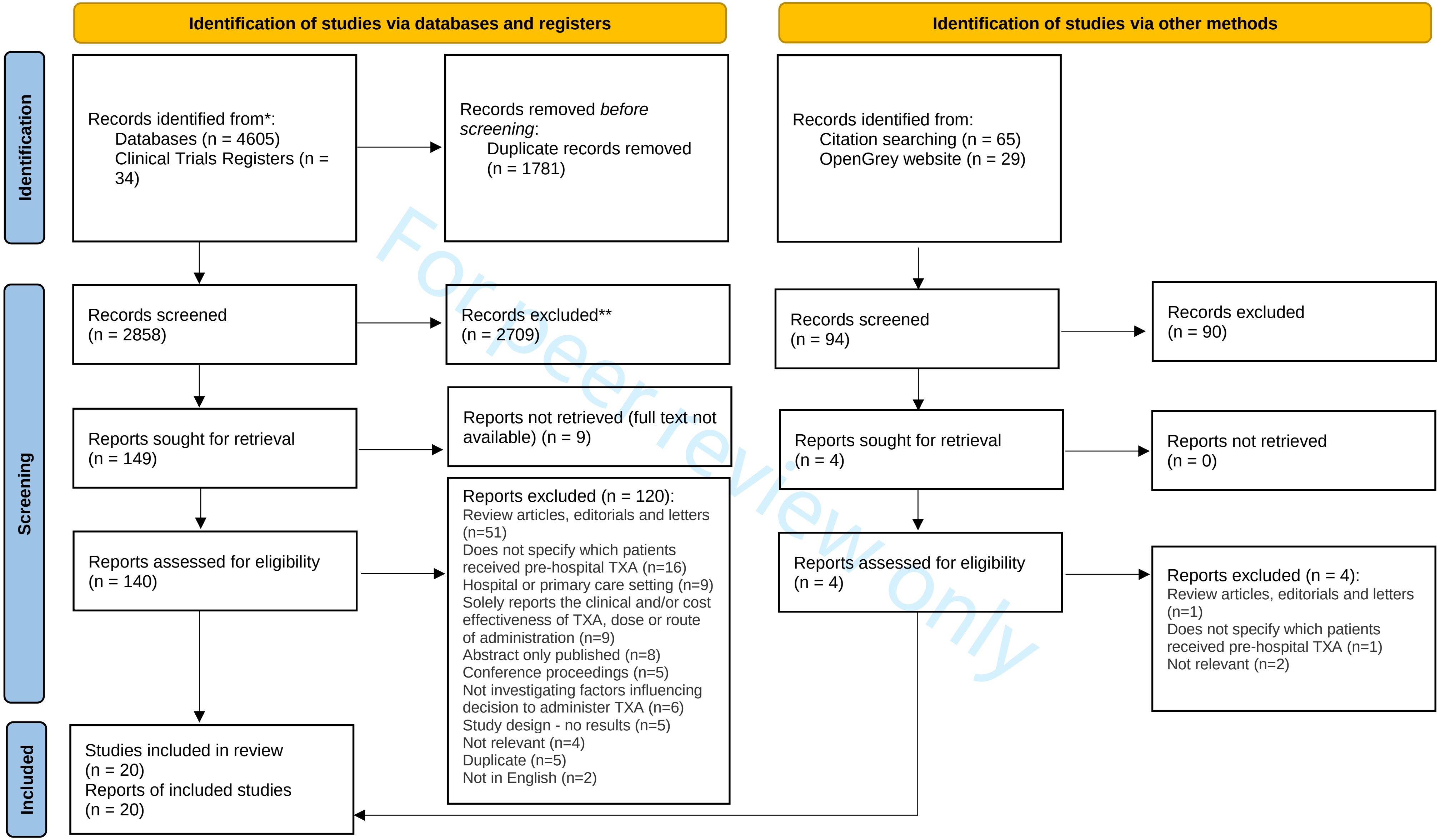

*Consider, if feasible to do so, reporting the number of records identified from each database or register searched (rather than the total number across all databases/registers).
**If automation tools were used, indicate how many records were excluded by a human and how many were excluded by automation tools.

From: Page MJ, McKenzie JE, Bossuyt PM, Boutron I, Hoffmann TC, Mulrow CD, et al. The PRISMA 2020 statement: an updated guideline for reporting systematic reviews. BMJ 2021;372:n71. doi: 10.1136/bmj.n71. For more information, visit: http://www.prisma-statement.org/

For peer review only

APPENDIX 5- Characteristics of included civilian studies (n=13).

First Author (date), country	Study design	Patient number	Data source	Dates and duration of data collection	Population	Factors influencing TXA administration
Bossers (2021), Netherlands	Observational mulitcentre cohort study	1827	Brain Injury: Prehospital Registry of Outcome, Treatments and Epidemiology of Cerebral Trauma (BRAINPROTECT) study database.	February 2012-December 2017, 5 years 10 months	Adult patients with severe TBI attended by HEMS	**Patient age** - patients receiving TXA older than those not receiving TXA (47 vs 45 years).<br><br>**Injury type, severity -** patients receiving TXA had higher Injury Severity Score (ISS) (27 vs 26), lower prehospital Glasgow Coma Score (GCS) (4 vs 5) and higher heart rate (98 vs 92).
El-Menyar (2020), Qatar	Retrospective observational, case control	204	Qatar National Trauma Registry	January 1 2017 to September 30 2018; 1 year 9 months	Adult trauma patients	**Resources** – patients did not receive TXA if critical care paramedics unavailable.
Goodwin (2021), UK	Qualitative interview study	18	UK paramedic interviews	2019	N/A	**Knowledge and skills** - inadequate training and a lack of knowledge of the effects of TXA or the evidence base behind its use and a lack of exposure to trauma patients were barriers to its administration.<br>**Resources -** a lack of time and staffing was a barrier to administration. Helicopter Emergency Medicine Services (HEMS) attendance was a barrier to some as they preferred to wait for a HEMS team member to administer it.<br>**Protocol -** guidelines felt restrictive or confusing. Disparity between paramedic, HEMS and doctor TXA protocols causes confusion. The drug preparation and administration route were seen as barriers to its use.<br>**Consequences and social influences-** the benefits of TXA were seen to outweigh the risks. TXA was seen to signal a major trauma patient. Fear of repercussion for administering TXA inappropriately. Opinion of colleagues seen to influence TXA administration.

For peer review only

						**Priorities** - TXA not seen as a priority, with administering fluids or distracting injuries often taking precedent. Three-hour window of administration may reduce the perceived urgency of administration. The stress associated with trauma jobs may lead to TXA being overlooked or deprioritised.<br>**Injury type, severity - r**isk of bleeding influenced administration including clinical observations and patient presentation. Participants found identifying patients at risk of bleeding difficult.<br>**Injury type, Mechanism of Injury (MOI) -** uncertainty over which injuries/conditions TXA is indicated for. MOI and type of injury were part of identify patients at risk of bleeding.<br>**Injury type, site** - less obvious bleeding including occult/internal haemorrhage was harder to identify.
Marsden (2019), UK	Retrospective service evaluation	661	TARN, local trauma registries and individual patient hospital records	January 1 2017 to December 31 2017; 1 year	Adult and paediatric trauma patients	**Injury type, MOI** – road traffic collisions more likely to receive TXA, falls less likely.<br>**Injury type, severity** – patients given TXA more likely to have heart rate and blood pressure suggestive of bleeding.<br>**Resources** – patients given TXA were more likely to be treated by a physician-led crew.
McQueen (2013), UK	Retrospective service evaluation	123	HEMS clinical database.	6 months - Dates not stated.	Major trauma patients attended by HEMS	**Protocol** - clinician judgement used to guide administration outside of the protocol.<br>**Injury type, MOI** – most TXA patients had multiple injuries.<br>**Resources** - time constraints on scene and the absence of a doctor as part of the HEMS crew meant TXA not given.
Neeki (2018), USA	Observational cohort study	724	Electronic medical record and trauma registry	March 2015 to July 2017; 2 years 4 months	Adult trauma patients	**Resources** - not all Emergency Medicine Service providers carrying TXA.
Ng (2018), Canada	Retrospective observational	117	British Columbia Trauma Registry and Royal Columbian Hospital records.	April 2012 to June 2015; 3 years 2 months.	Adult trauma patients	**Injury type, severity** – more patients receiving TXA had moderate (9-12) or severe (3-8) GCS scores than those not receiving TXA.
Nutbeam (2022), UK	Retrospective observational	216,364	Trauma and Audit Research	January 1 2017 to Dec		**Sex** - female patients less likely to receive TXA (OR 0.35, 95% CI, 0.33-0.36).

For peer review only - http://bmjopen.bmj.com/site/about/guidelines.xhtml

			Network (TARN) registry	31 2020; 4 years	Adult trauma patients	**Injury type, severity** - females less likely than males to receive TXA in all ISS categories.<br>**Injury type, MOI** - females less likely than males to receive TXA for all injury mechanisms except motor vehicle crashes.
van Wessem (2022), Netherlands	Prospective cohort study	234	Hospital data	November 2013; 7.5 years	Adult trauma patients with associated severe TBI	**Patient age** - patients receiving TXA younger than those not receiving TXA (42 vs 53 years).<br>**Injury type, severity -** patients needing pre-hospital intubation, urgent laparotomy or with more deranged physiology more likely to have received TXA.
van Wessem (2021), Netherlands	Population based Prospective cohort study	422	Hospital data	November 2013; 7 years	Adult trauma patients with associated severe TBI	**Patient age** - patients receiving TXA younger than those not receiving TXA (41 vs 51 years).<br>**Injury type, severity -** patients receiving TXA slightly more severely injured, had higher Abbreviated Injury Scale (AIS) Head scores and more often prehospitally intubated.
Vu (2013), USA	Case series	13	British Columbia Ambulance Service AirEvac and Critical Care Operation data system.	4 months - Dates unclear	Adult major trauma patients	**Priorities** - balancing critical interventions, resuscitation and short flight times meant some eligible patients did not receive TXA.
Wafaisade (2016), Germany	Retrospective observational	5765	ADAC Air Rescue Service prehospital database and German Trauma Society Trauma registry	January 1 2012 to December 31 2014; 3 years	Critically injured adult trauma patients	**Injury type, severity** - patients needing pre-hospital intubation or chest tube placement more likely to receive TXA.<br>**Injury type, site** - patients with AIS ≥3 for abdomen or extremities more likely to receive TXA. Patients with AIS ≥3 for head or thorax less likely to receive TXA.<br>**Age** - patients over 60 years old less likely to receive TXA.
Wong (2021), Canada	Retrospective observational	100	Hospital charts and British Columbia Trauma Registry	April 1 2016 to March 31 2017; 1 year	Adult trauma patients	**Age** - TXA group notably younger (38.2 years vs 49.1 years)<br>**Injury type, severity** - TXA group had higher mean ISS and more patients with hypotension.<br>**Resources** - more patients receiving TXA had a paramedic of higher certification level in attendance than those who did not receive TXA.

For peer review only

APPENDIX 6- Characteristics of included military setting studies (n=7).

First Author (date), country	Study design	Patient number	Data source	Dates and duration of data collection	Population	Factors influencing TXA administration
Benov (2019), Israel-Syrian Border	Retrospective observational	2339	Israel Defences Forces Trauma Registry	February 12 2013 to December 31 2017; 4 Years 10 months 19 days	Combat injuries, Syrian adult and paediatric refugees	**Protocol** – TXA for penetrating torso injury regardless of haemodynamic status. All patients receiving freeze-dried plasma with a known time of injury <3 hours received TXA.<br><br>**Injury type, MOI** – majority of TXA patients had penetrating injury.
Fisher (2019), Iraq and Afghanistan	Retrospective observational	28,222	The Department of Defence Trauma Registry.	January 2007 to August 2016; 9 years and 8 months.	Adult trauma patients	**Injury type, severity** – patients with higher ISS, tourniquet application or serious injuries to the thorax, abdomen, extremities and skin more likely to receive TXA.<br>**Injury type, MOI** - explosive injury and gunshot wounds were more likely to receive TXA. More likely to have explosive injuries and less likely gunshot wounds in TXA patients who had a tourniquet applied.<br>**Patient age** - amputation patients receiving TXA were younger than those not receiving TXA (22 vs 25 years).
Lipsky (2014), Israel	Retrospective observational	40	Israel Defences Forces Trauma Registry	December 2011 to February 2013; 1 year 2 months.	Adult trauma patients	**Protocol** – 30% of TXA administrations had no clear indication for TXA administration. Altered level of consciousness mistakenly categorised as a sign of haemodynamic instability.<br>**Priorities** – some non-administrations due to tactical limitations, resuscitation or to avoid delaying evacuation.
Mahalo (2021), Israel	Retrospective observational	1059	Israel Defences Force Trauma Registry and Israel National Trauma Registry	2006 to 2017; 10 years	Adult civilian and military trauma patients	**Injury type, severity** - TXA administration associated with analgesic treatment.
Nadler (2014), Israel	Retrospective observational	94	Israel Defences Forces Trauma Registry and hospital charts	December 2011 to August 2013; 1 year 8 months.	Adult civilian and military trauma patients	**Protocol** – more conservative protocol in the civilian service compared to the military service but higher proportion of patients given TXA outside of protocol (with clearance) by civilian service.

For peer review only

Nadler (2021), Israel	Retrospective observational	16529	Israel Defences Forces Trauma Registry and hospital charts	January 2006 to December 2018; 13 years	Adult civilian and military trauma patients	**Protocol -** New Clinical Practice Guidelines (indicating TXA at a heart rate of 130 instead of 110) introduced caused a significant decrease in the proportion of TXA administered. Only 22% of patients indicated for TXA received it.
Tsur (2020), Israel	Retrospective observational	3394	Israel Defences Forces Trauma Registry	January 1997 to December 2018; 11 years	Adult military trauma patients	**Injury type, site** – isolated neck injuries more likely to receive TXA than no-neck injuries.

For peer review only

PRISMA 2020 Checklist

Section and Topic	Item #	Checklist item	Location where item is reported
**TITLE**			
Title	1	Identify the report as a systematic review.	p.1
**ABSTRACT**			
Abstract	2	See the PRISMA 2020 for Abstracts checklist.	p.1-2
**INTRODUCTION**			
Rationale	3	Describe the rationale for the review in the context of existing knowledge.	p.2
Objectives	4	Provide an explicit statement of the objective(s) or question(s) the review addresses.	p.2
**METHODS**			
Eligibility criteria	5	Specify the inclusion and exclusion criteria for the review and how studies were grouped for the syntheses.	p.3. + Appendix 2
Information sources	6	Specify all databases, registers, websites, organisations, reference lists and other sources searched or consulted to identify studies. Specify the date when each source was last searched or consulted.	p.3 + Appendix 1
Search strategy	7	Present the full search strategies for all databases, registers and websites, including any filters and limits used.	Appendix 1
Selection process	8	Specify the methods used to decide whether a study met the inclusion criteria of the review, including how many reviewers screened each record and each report retrieved, whether they worked independently, and if applicable, details of automation tools used in the process.	p.3
Data collection process	9	Specify the methods used to collect data from reports, including how many reviewers collected data from each report, whether they worked independently, any processes for obtaining or confirming data from study investigators, and if applicable, details of automation tools used in the process.	p.3
Data items	10a	List and define all outcomes for which data were sought. Specify whether all results that were compatible with each outcome domain in each study were sought (e.g. for all measures, time points, analyses), and if not, the methods used to decide which results to collect.	p.3
	10b	List and define all other variables for which data were sought (e.g. participant and intervention characteristics, funding sources). Describe any assumptions made about any missing or unclear information.	p.3 + Appendix 3
Study risk of bias assessment	11	Specify the methods used to assess risk of bias in the included studies, including details of the tool(s) used, how many reviewers assessed each study and whether they worked independently, and if applicable, details of automation tools used in the process.	p.3
Effect measures	12	Specify for each outcome the effect measure(s) (e.g. risk ratio, mean difference) used in the synthesis or presentation of results.	N/A
Synthesis methods	13a	Describe the processes used to decide which studies were eligible for each synthesis (e.g. tabulating the study intervention characteristics and comparing against the planned groups for each synthesis (item #5)).	p.3
	13b	Describe any methods required to prepare the data for presentation or synthesis, such as handling of missing summary statistics, or data conversions.	N/A
	13c	Describe any methods used to tabulate or visually display results of individual studies and syntheses.	p.3
	13d	Describe any methods used to synthesize results and provide a rationale for the choice(s). If meta-analysis was performed, describe the model(s), method(s) to identify the presence and extent of statistical heterogeneity, and software package(s) used.	p.3
	13e	Describe any methods used to explore possible causes of heterogeneity among study results (e.g. subgroup analysis, meta-regression).	N/A
	13f	Describe any sensitivity analyses conducted to assess robustness of the synthesized results.	N/A
Reporting bias assessment	14	Describe any methods used to assess risk of bias due to missing results in a synthesis (arising from reporting biases).	p.3

For peer review only - http://bmjopen.bmj.com/site/about/guidelines.xhtml

PRISMA 2020 Checklist

Section and Topic	Item #	Checklist item	Location where item is reported
Certainty assessment	15	Describe any methods used to assess certainty (or confidence) in the body of evidence for an outcome.	p.3-4
**RESULTS**			
Study selection	16a	Describe the results of the search and selection process, from the number of records identified in the search to the number of studies included in the review, ideally using a flow diagram.	p.3-4 + Appendix 4
	16b	Cite studies that might appear to meet the inclusion criteria, but which were excluded, and explain why they were excluded.	N/A
Study characteristics	17	Cite each included study and present its characteristics.	Table 1 + Table 2 + Appendix 5 + Appendix 6
Risk of bias in studies	18	Present assessments of risk of bias for each included study.	p.3-4
Results of individual studies	19	For all outcomes, present, for each study: (a) summary statistics for each group (where appropriate) and (b) an effect estimate and its precision (e.g. confidence/credible interval), ideally using structured tables or plots.	Table 1 + Table 2 + Appendix 5 + Appendix 6
Results of syntheses	20a	For each synthesis, briefly summarise the characteristics and risk of bias among contributing studies.	p.4
	20b	Present results of all statistical syntheses conducted. If meta-analysis was done, present for each the summary estimate and its precision (e.g. confidence/credible interval) and measures of statistical heterogeneity. If comparing groups, describe the direction of the effect.	N/A
	20c	Present results of all investigations of possible causes of heterogeneity among study results.	p.3-10
	20d	Present results of all sensitivity analyses conducted to assess the robustness of the synthesized results.	N/A
Reporting biases	21	Present assessments of risk of bias due to missing results (arising from reporting biases) for each synthesis assessed.	p.3-4
Certainty of evidence	22	Present assessments of certainty (or confidence) in the body of evidence for each outcome assessed.	N/A
**DISCUSSION**			
Discussion	23a	Provide a general interpretation of the results in the context of other evidence.	p.10-12
	23b	Discuss any limitations of the evidence included in the review.	p.2, p.10-12
	23c	Discuss any limitations of the review processes used.	p.2, p.10-12
	23d	Discuss implications of the results for practice, policy, and future research.	p.10-12
**OTHER INFORMATION**			
Registration and protocol	24a	Provide registration information for the review, including register name and registration number, or state that the review was not registered.	p.2, p.14
	24b	Indicate where the review protocol can be accessed, or state that a protocol was not prepared.	p.2
	24c	Describe and explain any amendments to information provided at registration or in the protocol.	p.2
Support	25	Describe sources of financial or non-financial support for the review, and the role of the funders or sponsors in the review.	p.2, p.13

For peer review only
For peer review only - http://bmjopen.bmj.com/site/about/guidelines.xhtml

PRISMA 2020 Checklist

Section and Topic	Item #	Checklist item	Location where item is reported
Competing interests	26	Declare any competing interests of review authors.	p.13
Availability of data, code and other materials	27	Report which of the following are publicly available and where they can be found: template data collection forms; data extracted from included studies; data used for all analyses; analytic code; any other materials used in the review.	Available on request.

From: Page MJ, McKenzie JE, Bossuyt PM, Boutron I, Hoffmann TC, Mulrow CD, et al. The PRISMA 2020 statement: an updated guideline for reporting systematic reviews. BMJ 2021;372:n71. doi: 10.1136/bmj.n71

For more information, visit: http://www.prisma-statement.org/

For peer review only
